# Study of Endocrine-Disrupting Chemicals in Infant Formulas and Baby Bottles: Data from the European LIFE-MILCH PROJECT

**DOI:** 10.3390/molecules29225434

**Published:** 2024-11-18

**Authors:** Francesca Nuti, Feliciana Real Fernández, Mirko Severi, Rita Traversi, Vassilios Fanos, Maria Elisabeth Street, Paola Palanza, Paolo Rovero, Anna Maria Papini

**Affiliations:** 1 Interdepartmental Research Unit of Peptide and Protein Chemistry and Biology (Peptlab) and Centre of Competences in Molecular Diagnostics and Life Sciences (MoD&LS), University of Florence, 50019 Sesto Fiorentino, Italy; francesca.nuti@unifi.it (F.N.); feliciana.realfernandez@iccom.cnr.it (F.R.F.); paolo.rovero@unifi.it (P.R.); 2 Department of Chemistry “Ugo Schiff”, University of Florence, 50019 Sesto Fiorentino, Italy; mirko.severi@unifi.it (M.S.); rita.traversi@unifi.it (R.T.); 3 Institute of Chemistry of Organometallic Compounds, National Research Council (ICCOM-CNR), 50019 Sesto Fiorentino, Italy; 4 Section of Neonatal Intensive Care Unit, Department of Paediatrics, Puericulture Institute and Neonatal Section, Azienda Mista and University of Cagliari, 09124 Cagliari, Italy; vafanos@tiscali.it; 5 Department of Medicine and Surgery, University of Parma, 43126 Parma, Italy; mariaelisabeth.street@unipr.it (M.E.S.);; 6 Unit of Pediatrics, University Hospital of Parma, 43126 Parma, Italy; 7 Behavioral Biology Laboratory, University of Parma, 43124 Parma, Italy; 8 Department of Neurosciences, Psychology, Drug Research and Child Health (NeuroFarBa), University of Florence, 50019 Sesto Fiorentino, Italy

**Keywords:** endocrine-disrupting chemicals, biomarkers, infant formula, phthalates, bisphenols, baby bottles, heavy metals

## Abstract

Exposure to endocrine-disrupting chemicals (EDCs) is inevitable, and growing scientific evidence indicates that even very low doses can negatively impact human health, particularly during pregnancy and the neonatal period. As part of the European project LIFE18 ENV/IT/00460, this study aims to identify the presence of EDCs in 20 infant formulas (both powdered and liquid) and the release from baby bottles and teats. Particularly, sensitization of young people and future parents towards the potential harmful effects of EDCs could significantly help to reduce exposure. Seven different UPLC-MS/MS methodologies and one ICP-AES were set up to quantify already assessed and suspected EDCs among 85 different chemicals (bisphenols, parabens, PAHs, phthalates, pesticides, herbicides and their main metabolites, PFAS, and metals). Results showed that in 2 out of 14 baby bottles, only anthracene and phenanthrene of the group of PAHs were released (10.68–10.81 ng/mL). Phthalates such as mono-ethyl phthalate (MEP) were found in 9 of 14 samples (0.054–0.140 ng/mL), while mono(2-ethyl-5-oxohexyl) phthalate (MeOHP) appeared in 2 samples (0.870–0.930 ng/mL). In accordance with current EU regulations, other chemicals were not detected in baby bottles and teats. However, bisphenols, parabens, PAHs, phthalates, PFAS, and metals were detected in infant formula, emphasizing the need for continued monitoring and public health interventions.

## 1. Introduction

Exclusive breastfeeding during the first 6 months of life is highly recommended by all pediatric scientific societies and by the World Health Organization (WHO), and breastfeeding is encouraged also while introducing complementary foods, and up to 2 years of age or longer [1,2]. Infant formula (IF) or combination feeding becomes necessary if maternal milk is insufficient or maternal circumstances do not allow breastfeeding.

A few studies have shown that pollutants are detectable in both breast and formula milk, posing questions on the possible consequences of exposures in utero, and early childhood exposures can have lifelong consequences on health and diseases [3,4,5]. Diet and lifestyle are important factors related to exposure to environmental pollutants, among these endocrine-disrupting chemicals are included.

In 2018, the European Food Safety Authority (EFSA) and European Chemicals Agency (ECHA) guidance document described the substances with endocrine-disrupting properties in pesticides and biocides [6]. According to this latest document, an endocrine-disrupting chemical (EDC) is a substance showing an adverse effect in an intact organism, having an endocrine mode of action, i.e., capable of altering the function(s) of the endocrine system, and the adverse effect is a consequence of the endocrine mode of action. EDCs acting as hormone receptor agonists or antagonists interfere with post-receptor signal transduction, hormone synthesis, transport, distribution, and clearance [7,8,9,10,11,12]. Their bioactivity is triggered by different mechanisms with nuclear receptors. Adsorption of EDCs by inhalation, ingestion, and skin permeation in specific periods of life can generate critical windows of exposure. In particular a prenatal, perinatal, or early life exposure is associated with a very high risk of developing diseases in later periods of life, mainly through epigenetic changes, altering development phases. Puberty and adolescence are also considered critical periods of life for EDCs exposure [13,14]. In this context, monitoring the substances that might represent a health threat, that interfere with hormonal pathways, and that are related to the environment, food, and consumer products, is a growing need. The group of molecules identified as endocrine disruptors is highly heterogeneous. Synthetic chemicals used in industrial processes as plastics (bisphenols), polycyclic aromatic hydrocarbons (PAHs), plasticizers (phthalates), pesticides (chlorpyrifos, glyphosate), insecticides (pyrethroids), and preservatives of cosmetics and pharmaceutical products (parabens) are among the possible release compounds [15]. Perfluoroalkyl substances (PFASs) are a numerous group of compounds characterized by a chemical resistance and are used in a variety of industrial applications and consumer products. EDCs are known to have effects on puberty and growth, thyroid function and neurodevelopment [16], the immune system [17], metabolism [14], obesity [18], and fertility [19].

Since IFs are intended to serve as a substitute for breast milk in infants who cannot be breastfed, their composition should meet specific nutritional requirements to promote the regular growth and development of babies. Thus, IFs should only contain components in such amounts that provide a nutritional purpose or other benefits. The inclusion of unnecessary components, or unnecessary amounts of components, may alter metabolic and/or other physiologic functions of the infant. For this reason, minimum and maximum levels of nutrient contents in IFs are suggested with the aim to provide safe and nutritionally adequate IF products. Regulations and directives for restricting EDCs and persistent organic pollutants in IFs have been reviewed by Hatzidaki et al. including risk assessment studies [20].

In this context, metals and metalloids are present in IFs both because they are added for nutritional purposes and as inorganic pollutants. The metal uptake by newly born children depends on bioavailability from milk diets and can affect physical and cognitive development irreversibly [21]. Thus, they represent a concern, although studies in this regard are still relatively scarce [22]. Specific regulations impose minimum and maximum values for some essential elements (Na, K, Cl, Ca, P, Mg, Fe, Zn, Cu, I, Se, Mn, and F) and maximum levels for some inorganic contaminants (e.g., As, Cd, and Pb) [23]. In terms of health hazards, Cd, Cr, Mn, Ni, Pb, and Zn are particularly relevant because of their bio-accumulation in vital organs, long-term persistence in the human body, and possible serious negative effects on infants’ health [24]. Although current legislation devoted to IFs imposes limits for the above mentioned metals, today, there are no guidelines concerning Aluminium, and recommendations remain inadequate. A number of publications clearly elucidate Al contamination [25,26], possibly due to ingredients, packaging, and processing [27].

Although most toxic agents are hazardous in high doses, the human health risk associated with EDCs concerns continuous low dose exposures. Maternal exposure to EDCs during pregnancy results in fetal exposure through the placenta, and neonatal exposure can continue through breast milk and/or formula milk. Exposure to EDCs is unavoidable and increasing scientific evidence has shown that they have negative effects on growth, bone, adipose tissue, metabolism, puberty, and fertility, and they cause a predisposition to developing cancer. IFs can potentially contain harmful contaminants and residues considering the source of the raw material, production sites [21], and possible contaminants from the packaging. Although external contaminants are continuously monitored and certified, and regulated by legislations that have established maximum limits and guide values for newborns’ safety, an increasing number of environmental chemicals have been measured in formula besides breast milk [28], eliciting a need for improved methodologies for assays and continuous biomonitoring and an adjustment of safety limits of exposure.

Furthermore, in this context, increased public awareness is essential for the perception of the risk of health damage from exposure to EDCs. Particularly, sensitization of young people and future parents towards the potential harmful effects of EDCs could significantly help to reduce exposure. The LIFE18 ENV/IT/00460—Life MILCH project entitled “Mother and Infant dyads: Lowering the impact of endocrine disrupting chemicals in milk for a Healthy Life”, supported by the European Community, aims at evaluating exposure to a number of EDCs and to their metabolites of mothers at the end of pregnancy, and of their newborns through their detection in serum, urine, and breast milk. A subsequent follow-up at 1, 3, 6, and 12 months of infant life is ongoing with a special focus on the relationships among the possible sources of EDCs, the neurodevelopment, growth, distribution of adipose tissue, and pubertal stages.

The current study aimed at evaluating the amount of EDCs present in the IFs that were used when needed. Eight different methodologies to quantify 85 different chemicals (bisphenols, parabens, phthalates, pesticides, herbicides, and their main metabolites, PFAS, and metals) were set up. We moved on from the existing methods in the literature, improving and validating the modified protocols. Moreover, besides the IFs, we evaluated the release of EDCs from the IF containers, bottles, and teats.

## 2. Results and Discussion

EDCs can be present in IF and/or in its containers. The presence of EDCs in IF was widely documented in the literature, but often such studies focused on a single group or at most two groups of EDCs. Herein, we reported a systematic study to quantify eight different groups of EDCs: 4 different bisphenols, 11 different polycyclic aromatic hydrocarbons (PAHs), 14 phthalates (diesters and corresponding monoesters), 7 parabens, 3 polar pesticides, 2 pyrethroids and chlorpyrifos, 27 PFAS, and 16 metals to establish a wide exposure risk for infant growth. Assessed and potential endocrine-disrupting chemicals selected for the study are shown in the Supplementary Materials (Table S1). With this aim the present study consists of the following steps: (i) the development of the analytical method for each EDC group quantification, including the sample extraction procedure optimization, (ii) the analysis of the background considering that these chemicals are ubiquitous, and (iii) obtaining an overview of children exposure during feeding time.

Considering that EDCs can be present not only in IF but also in their containers, seven of the most commonly available baby bottles, and eight feeding bottles shipped directly from intensive natal therapy units (with and without their corresponding teats) were analyzed. Their release of molecules of the different EDC groups was evaluated.

### 2.1. Performance of the Assays for Simultaneous Determination of EDCs

The analytical methods to identify and quantify the seven different groups of assessed or suspected EDCs were optimized independently by UPLC-MS/MS using the specific standards (Table S2). Thus, sensitive and reliable analytical methods allowed the determination of the different compounds. Starting from previously reported methodologies, the quality control (QC) and quality assurance (QA) for each analytical procedure were evaluated. In particular, operational upgrades were applied to quantify an increased number of compounds, reducing the analysis time and/or changing the solvent system. The efficiency of each method was evaluated considering the linearity, range, accuracy, precision, limit of detection (LOD), and limit of quantification (LOQ). All parameters obtained for each compound were demonstrated comparable to those previously reported in the literature. In particular, the precision and accuracy were assessed by the intra- and inter-assays obtaining values within acceptable ranges (see Section 3).

#### 2.1.1. Bisphenols

Bisphenol A (BPA) is used as an additive to make clear and hard polycarbonate plastics such as plastic bottles and sippy cups. BPA used in plastic containers can migrate to food, particularly at high temperatures [29]. For this reason, BPA is currently prohibited both in formula milk and in baby food containers [30,31], and the European Food Safety Authority (EFSA) has decreased the tolerance daily intake (TDI, the amount of a substance that can be consumed daily over a lifetime without presenting an appreciable risk to health) from 4 ug/kg in 2015 to 0.2 ng/kg in 2023 [32]. But these restrictions open the way to other similar structural analogues such as bisphenol S (BPS) and bisphenol F (BPF). For this reason, in this study, we evaluated not only the presence of BPA, but also BPS and BPF, which are not regulated by the EU. Moreover, given that in the literature, there is a lack of information on the toxicity levels and their combined effects information on new bisphenol derivatives, the bisphenol F diglycidyl ether (BPFDGE) was also monitored.

To separate four bisphenols, an UPLC–MS/MS analysis was set up starting from the previous method reported by van der Meer et al. [33]. The analytical method is described in the Supplementary Materials (Table S3). No significant matrix effect differences were observed in the analysis of the bisphenols.

#### 2.1.2. Parabens

Parabens are extensively used as preservatives in cosmetics, pharmaceuticals, and food products, including beverages. They stop mold and bacteria growth in baby skin care products that potentially harm the baby. The former EC Scientific Committee for Food evaluated the parabens in 1994 and allocated their acceptable daily intake at 0–10 mg/kg b.w. (milligrams/kilogram body weight); for the sum of methyl, ethyl, and propyl p-hydroxybenzoic acid esters and their sodium salts, the limit was further confirmed by EFSA in 2004 [34].

Although the occurrence of parabens in humans has been reported, few investigations about milk and IF are available [35]. In order to quantify the level of parabens in the IF, we proposed to separate seven parabens with an efficient LC–MS/MS method starting from the previous method reported by Dualde et al. [36].

The UPLC–MS/MS analytical method used to separate seven parabens is presented in the Supplementary Materials (Table S4). No significant matrix effect differences were observed in the analysis of the parabens.

#### 2.1.3. Polycyclic Aromatic Hydrocarbons (PAHs)

In nature, more than 100 PAHs exist. In the present study, the 11 PAHs listed by the Commission Regulation (EC) No. 1881/2006 were selected and reported in Table S1. Some of which include the following: benzo(a)pyrene (BPA), dibenzo(a,h)anthracene, benzo(a)anthracene, benzo(b)fluoranthene, benzo(k)fluoranthene, chrysene, and indene (1,2,3-cd) perylene have been classified by the Agency for Research on Cancer (IARC) as carcinogenic to humans or possibly carcinogenic to humans. The level of contamination of IF by PAHs is likely to depend on the geographic conditions where the milk was collected and the condition of the farm animals [37,38].

According to the EU Scientific Committee on Food, benzo(a)pyrene can be used as a marker of occurrence and effect of carcinogenic PAH in food, including also benzo(a)anthracene, benzo(b)fluoranthene (BBF), benzo(k)fluoranthene (BKF), benzo(g,h,i)-perylene, chrysene, and dibenzo(a,h)anthracene. Further analysis of the relative proportions of the different PAHs in food is essential to include all PAHs and not only benzo(a)pyrene as a marker [39].

The quantitative analysis of 11 PAHs was carried out using an UPLC–MS/MS method presented in the Supplementary Materials (Table S5). As EFSA suggested, the quantification of BBF, BKF, and BAP was reported as sum of concentrations of the three analytes (ΣPAHs).

#### 2.1.4. Glyphosate and Its Metabolites

Glyphosate is widely used in herbicide products and its use has been authorized for 10 years by the European Commission in extensive agriculture following a safety evaluation.

Many laboratories are studying an efficient method to separate and identify at trace levels glyphosate, its major metabolite (aminomethyl)phosphonic acid (AMPA), and glufosinate ammonium considering their dramatic increase as herbicides [40]. We propose a new, fast, and simple extraction method to remove all products present in the milk matrix that interferes with the analysis, followed by liquid chromatography coupled with the determination of triple quadrupole mass spectrometry to quantify glyphosate, glufosinate, and AMPA. Moreover, we also propose a fast and efficient chromatographic separation protocol in only 8 min without molecules derivatization, overcoming the previously described protocols, which reported a chromatographic separation using derivatization methodologies. Thanks to the new column Raptor Polar X characterized by unique hybrid phase that balances HILIC and ion exchange retention modes, we can separate the polar compounds as glyphosate and its metabolites applying the classical mobile phase water/ACN with formic acid. The eluent for separation was H_2_O/ACN with 0.5% of HCOOH and the ESI-MS is in the MRM negative mode.

In order to avoid ionization suppression, a passivation procedure of the LC system was performed before real sample analyses. A total of 10 full loop injections of the 10 mM medronic acid were performed without column, with the mobile phases directed to waste instead of the mass spectrometer and 5 full loop injections (2 µL) with the column was performed using analytical method conditions improving peak shape. After this process, all polar phosphorylated pesticides exhibited sharp and symmetrical peaks for the subsequent injections.

With all these precautions, the described method allowed us to obtain calibration curve plots independently for glyphosate, glufosinate, and AMPA with optimal correlation coefficients (R^2^ > 0.99) generated with 10 serial dilutions of the stock solution. The intra-assay coefficients of variability (CV) were ≤10% and inter-assay CV were ≤12% for all analytes at two QC levels. Recoveries ranged from 84 to 104%. LODs and LOQs for each chemical are summarized in the Supplementary Materials (Table S6). Herein, a rapid and sensitive method without derivatization was described for quantification of glyphosate and its metabolites.

#### 2.1.5. Phthalates (Diesters and Corresponding Monoesters)

Phthalates are classified as EDCs and have been linked to adverse health effects, particularly in relation to early life exposures. Inhalation, ingestion, and skin contact are the major exposure via, even if recent studies demonstrate that a majority of exposure is probably food related [41] and correlate with metabolic disorders [16,18]. Moreover, diesters can migrate into food from plasticized PVC materials such as tubing typically used in the milking process, lid gaskets, food-packaging films, and gloves used in the preparation of foods [42].

Among all the possible phthalates described in the literature, in this study, 14 compounds were selected: six diesters and their respective eight monoester metabolites. Monoesters phthalates originate when the corresponding diesters enter the organism and are hydrolyzed and then further oxidized through complex pathways.

To separate 14 phthalates, LC—MS/MS analysis was set up starting from our previously described method [43] and the one reported by van der Meer et al. [44]. Modifications to this methodology were applied. Particularly, the analytical method developed to separate and quantify 14 different phthalates is a 22 min method, longer than those described by van der Meer et al., but with a single injection. Using the biphenyl column, the di- and monoesters were well separated, even those with similar characteristics, such as DEHP and DnOP or MEHP and MnOP. Electrospray ionization with a negative switching mode was adopted. The developed method uses another column. In fact, one of the most difficult problems in accurately quantifying trace levels of phthalates in a sample, is a background contamination. Since phthalates, given their extensive use and their persistence, are ubiquitous environmental contaminants, they are present as contaminants in almost all laboratory equipment, solvents, and laboratory air. To overcome the contaminations of phthalates and have a good separation between the background phthalate response from the phthalates in the sample, a second column (isolator column) was inserted in the method. In this way, the phthalates from the sample elute later than the background phthalates. Hence the quantification of phthalates in the sample is more accurate and the determination is more sensitive.

Analytical parameters for each chemical are presented in Table S7. The problem of the matrix effect observed for analysis of the phthalates was discussed in Section 2.2. Control of Contamination.

#### 2.1.6. Pyrethroids and Chlorpyrifos

The broad use of insecticides in intensive agriculture has increasingly sparked the interest and the need to develop analytical methods capable of detecting the intricacies of these substances in food and in particular in baby food.

Pyrethroids constitute one of the major group of insecticides, largely applied because they present non-systemic effects in plants. They are also found to be effective as a contact insecticide and stomach poison because of their anti-feeding action towards insects and arachnids. In this study, cypermethrin and cyfluthrin pyrethroids were chosen and an efficient method was developed to determine their concentration in formula milk. Moreover, the herbicide chlorpyriphos has been included in the pyrethroid mixture in order to develop an efficient protocol for their quantification. Starting from the previous method reported by Tran et al. [45], the proposed protocol is summarized in the Supplementary Materials (Table S8).

#### 2.1.7. Perfluoroalkyl Substances (PFASs)

Perfluoroalkyl substances (PFASs) are a very large class of compounds (>30,000) used in various consumer and industrial product sectors thanks to their high thermal, chemical, and enzymatic resistance. Over the years, they have bioaccumulated in the environment and humans and animals have assimilated these substances mainly through water and air. In fact, unlike other compounds, PFASs bind with high affinity to proteins so their half-time release is very long (5–8 years) [46,47,48]. In recent years, the European Commission has shown considerable interest in these fluorinated compounds and its particular interest is aimed at studying their effects on human health [49]. In particular, the scientific community is investigating their possible classification as EDCs [50,51]. In particular, EFSA has classified PFOS as such [52].

In 2022 the European Union recommended monitoring the presence of four PFAS in foods, PFOS, PFOA, PFNA, and PFHxS, and also determined the limits allowed in infant foods.

On this occasion, the European committee strongly advised to monitor the presence of other fluorinated compounds in foods [53]. In fact, the EU commission commits to phasing out all PFAS, allowing their use only in the irreplaceable cases essential to society [54]. Many recent studies are highlighting that infants can be contaminated with PFAS through breast milk [55,56]. The values found today in breast milk are slightly higher than in formula milk [57].

Herein, we investigated the presence of 27 PFASs in IF, following the extraction and HPLC–MS/MS methodologies as previously described [58]. Analytical data are summarized in Table S9.

### 2.2. Control of Contamination

After setting up a sensitive and reliable analytical method to separate and identify each compound in the seven EDCs groups, we investigated with all the methodologies employed, the background contamination due to the system, solvents, reagents, glassware, microfilters, plastic containers, and consumables for sample storage. In order to avoid and check the contamination mainly due to phthalates, parabens, and bisphenols, some preliminary procedures were taken before starting the quantification process and during sample collection and treatment.

The first step was based on the identification and evaluation of the potential contaminations in the labware materials, selecting from the market the ones with minor release of EDCs. In particular, there are potential origins of contaminations in reagents, solvents, and plastic containers used during all the quantification process. Moreover, the analysis of the entire laboratory stuff, i.e., gloves, plastic containers, and consumables for sample storage, have been tested to evaluate eventual EDCs release. Contamination from solvents and reagents during the analysis was monitored through the area response of reagent blank, procedural blank, and mobile phase injections.

The second step was based on the identification of the best consumables for sample storage. For this purpose, pure water was stored for 3 months in different plastic containers to quantify the contamination. After repeated quality controls, the best storage containers were selected of polypropylene (PP) or polyethylene (PE) composition.

The third step was based on the removal of some particular phthalates and PFAS contributing to a background contamination during phthalates and PFAS evaluation. In this regard, we placed the isolator column in-line between the solvent mixer and the injector to isolate the background contamination and to delay interferences with sample-containing phthalates to be able to obtain low detection limits [59].

### 2.3. Sample Pre-Treatment

**Figure 1:**
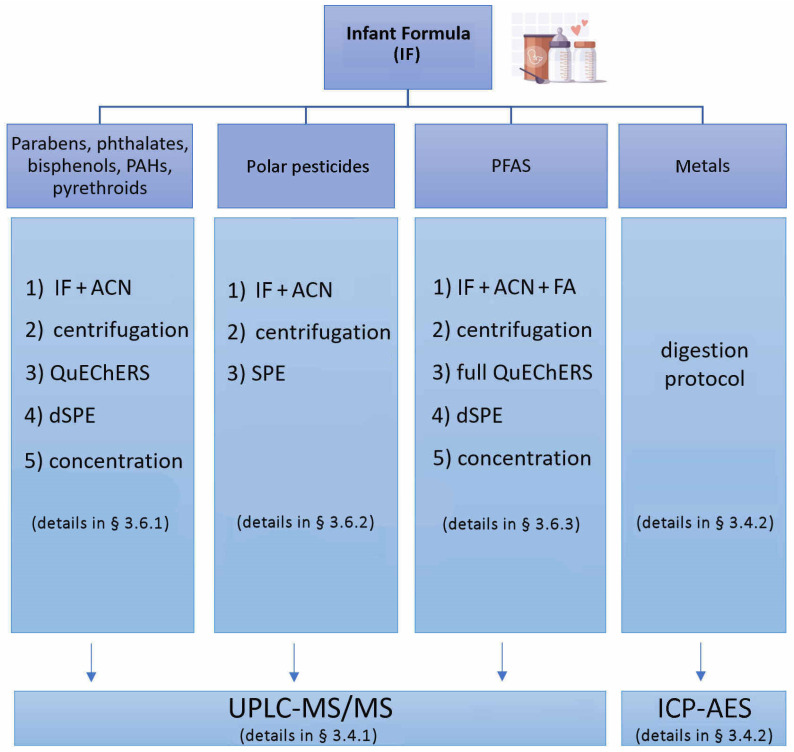
Pre-treatments of Infant Formula samples.

An exhaustive clean-up of the sample extract is necessary before the UPLC–MS/MS analysis. For this reason, four specific and sensitive protocols for milk samples pre-treatment have been set up and applied (Figure 1). To remove the protein content (about 3.5%) acetonitrile was used in the extraction procedure as extractant solvent thanks to its excellent ability for protein precipitation [60]. The content of lipids (about 4–6%), mostly represented by esters of fatty acids, is another main interference that can decrease the column lifetime and may bring non-negligible matrix effects. Before the UPLC–MS/MS of IF sample, remaining proteins, fats, and other components interfering with the analysis and quantification of the different bisphenols, PAHs, phthalates, parabens, pyrethroids, and chlorpyrifos, a standard QuEChERS method was applied. Then, the efficiency based on recoveries of extracted compounds was also evaluated.

In the case of glyphosate and its metabolites, the recommended and validated method by EU (EURL-SRM version 8.1, 2015) for extraction based on the QuPPe method showed low recoveries when applied to the IF. For this reason, herein, we set up a new protocol filtering the aqueous solution after protein precipitation with ACN through a STRATA-X SPE plate to clean up the lipid content from the sample. Thanks to this new protocol, we obtained high recoveries for glyphosate, glufosinate, and AMPA (ranging from 70 to 110%). The new protocol selected in this study presented several advantages such as reduced time for extraction, low cost, and operational simplicity.

The full QuEChERS extraction and dSPE cleanup for PFAS compounds is outlined in the described protocol [44] and summarized in the experimental part.

### 2.4. Quantification of Assessed and Suspected EDCs in Baby Bottles

Results showed that no EDCs of bisphenols, parabens, pesticides, PFAS, and pyrethroids were detectable in baby bottles and teats. These results are in accordance with the presence of BPA-free, PVC-free, and DEHP-free labels in most part of the baby bottles tested.

Anthracene and phenanthrene within the PAHs group were detected in 2 out of 14 samples (14% of samples; levels from 10.68 to 10.81 ng/mL, mean 10.75 ng/mL). Moreover, the analyses of phthalates showed the presence of mono-ethyl phthalate (MEP) in 9 out of 14 samples (64% of samples; levels from 0.054 to 0.14 ng/mL, mean 0.073 ng/mL) and MeOHP in 2 out of 14 samples (14% of samples; levels from 0.87 to 0.93 ng/mL, mean 0.90 ng/mL). On the other hand, samples flowed through their corresponding teats did not present increased concentrations. Thus we can conclude that teats did not contribute to increase the release of EDCs.

The EDCs were not detected in any of the sterile feeding bottles used in the neonatal intensive care units involved in the MILCH project, but only in those purchased from the local pharmacies selected for the present study. Additionally, one worn baby bottle with its nipple that a mother used daily to feed the baby, was also tested. No trace of EDCs was found in the bottle used repeatedly for breastfeeding. This result is in line with a previous study by Siddique et al. in which it was reported that the repeated use of the baby bottles did not increase the leaching of chemicals [61], confirming the suitability and strength of the material of the baby bottle. Moreover, no chemical compounds were found in the kit of the manual breast pump.

### 2.5. Quantification of Assessed and Suspected EDCs in Infant Formula

**Figure 2:**
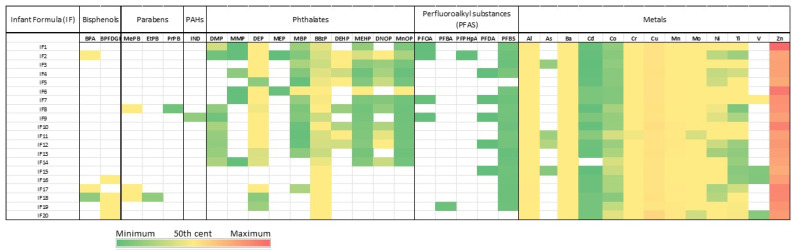
Concentrations expressed in ng/mL of assessed and suspected EDCs in infant formula milk. Using the color spectrum, the green color indicates minimum concentration, the yellow color indicates the 50th percentile, and the red color the maximum concentration.

Applying the previously described methodologies, we selected 20 commercially available infant milk including 11 formula type 1 and 9 formula type 2 and the findings are summarized in Table 1 and in Figure 2.

Among the bisphenols, detectable levels of BPA and BFDGE were observed. In particular, BPA was found in 3 out of 20 samples (15%) and BFDGE was found in 4 out of 20 samples (20%).

Previously published Italian studies on IFs reported the presence of BPA. In particular, Cirillo et al. declared to detect BPA levels in 60% of milk samples (levels between 3 and 375 pg/mL, median 15 pg/mL) [62]. Also E. Ferrer et al. declared to find BPA levels on 2 out of 5 IFs (levels from 0.07 to 1.29 mg kg^−1^) in a comparative Spanish-Italian study on IFs [63]. In the meantime, regulation of BPA has been more restricted; the EU Commission Regulation 2018/213 established that no migration of BPA shall be permitted from varnishes or coatings applied to materials and articles specifically intended to come into contact with IF, follow-on formula, baby food of infants and young children, or milk-based drinks and similar products specifically intended for young children. No further regulation has been found for other substances in the class of bisphenol-based molecules despite other bisphenols are under investigation.

Regarding parabens, MePB was detected in 3 out of 20 samples (15%), EtPB was detected in 1 out of 20 samples (0.5%), and PrPB was detected in 1 out of 20 samples (0.5%).

PAHs were not present except indenol (IND) that was quantifiable in 1 out of 20 samples (0.5%).

In a previous study focused in Southern Italy, Santonicola et al. reported that the levels of PAHs detected in commercial milk were 53.68 mg kg^−1^. In some cases, the ΣPAHs level exceeded the allowed limit of 1 mg kg^−1^, which could be linked to the presence of petrogenic and pyrolytic environmental sources, i.e., the incineration of waste present in the area surrounding the residence. This is the main source of exposure for infants during breastfeeding: through the exposure of mothers residing in some areas of Southern Italy [64].

Among phthalates, DMP was detected in 9 out of 20 samples (45%), DEP was detected in 17 out of 20 samples (85%), BBP was detected in 20 out of 20 samples (100%), DnOP was detected in 10 out of 20 samples (50%), DEHP was detected in 8 out of 20 samples (40%), MMP was detected in 6 out of 20 samples (30%), MEP was detected in 2 out of 20 samples (10%), MBP was detected in 14 out of 20 samples (70%), MnOP was detected in 14 out of 20 samples (70%), and MEHP was detected in 14 out of 20 samples (70%).

By analyzing the data obtained in this study, it can be confirmed that there is a phthalate pollution in the milk used for feeding, in fact most of the diesters and some monoesters selected for the study have been quantified. Breaking it down, BBP and DEP are the most frequently encountered analytes followed by DEHP and DnOP. In addition, the following hydrolyzed monoesters were also identified: MMP, MEP, MBP, MEHP, and MnOP. The oxidative monoesters MEHHP and MEOHP in IF were not detected.

Other Italian studies on IF have shown contamination of phthalate diesters and monoesters highlighting an extent of phthalate diester contamination, consideration supported in the broad data reported in the literature on IFs [65,66]. In particular, we confirmed that long-chain alkyl phthalates (DEHP and DnOP) and their respective metabolites (MEHP and MnOP) were detected in most of the samples, as evidenced in other studies.

No residues of chlorpyrifos, glyphosate or its main metabolites glufosinate and AMPA, and pyrethroids were detected in the 20 IFs tested.

Among the 27 PFASs studied, detectable levels of PFOA, PFBA, PFPHpA, PFDA, and PFBS were found. Only perfluorooctanoic acid (PFOA) of the four EU-regulated compounds was detected in 4 out of 20 samples (20%). Moreover, three perfluoroalkyl carboxylic acids were detected: PFBA in 1 out of 20 samples (5%), PFPHpA in 1 out of 20 samples (5%), and PFDA in 5 out of 20 samples (25%). Perfluoroalkane sulfonic acid PFBS was detected in 18 out of 20 samples (90%). Lakind et al. reviewed publications reporting PFAS in IF and concluded that PFAS measurement data for IF were sparse and the reported mean PFOA concentrations were slightly above the children’s drinking water screening values [57]. In fact, no PFAS residues were found in the European studies reported in the review. To the best of our knowledge, just target analytes PFOS and PFOA were previously detected in Italian cow milk with measured contaminations up to 97 ng/L and 32 ng/L, respectively [56].

Concerning metals, Bi, Pb, and Tl showed concentrations below the reported LOQ in all the analyzed samples. As and V were observed at concentrations above the LOQ only in 4 samples out of 20 and only 1 of those, both exceeded the LOQ simultaneously. All the other measured metals (Al, Ba, Cd, Co, Cr, Cu, Mn, Mo, Ni, Ti, Zn) showed concentrations well above the LOQ in all the samples.

Among the elements detected, Cu was present at the highest concentrations, followed by Mn and Al, with the latter showing comparable mean and median values. The lowest concentrations were observed for Cd, As, and Co. Cu and Mn are both essential nutrients for infants’ growth and toxic elements. Cu is needed for cellular metabolism in enzymatic and non-enzymatic systems [67] and its deficiency may cause growth impairment, neutropenia, anemia, and increased risk of infection [68]. Nevertheless, excessive chronic exposure to Cu may cause acute gastrointestinal symptoms, such as abdominal pain, vomiting, and diarrhea [69]. Serious concerns have recently been raised about relatively high Mn exposures and possible adverse effects on child neurodevelopment, with a particular attention to attention deficit hyperactivity disorder (ADHD) [70].

The presence of Al and other metals such as As and Cd (although to a lesser extent) in milk can be ascribed to environmental contamination (soil or grasslands), milk production or storage, and shipment to industrial plants [21].

### 2.6. Estimated Dietary Exposure in Infants at 1 Month of Age

To better evaluate the exposure of infants to EDCs due to daily consumption of the powdered IF type 1, the estimated daily intake (EDI) was calculated for each group of EDCs at 30 days after birth. The newborn body weight was assumed considering the WHO growth curves [71]. The EDI was calculated and reported in Table 2, comparing the hypothetical exposure with the regulatory limits currently available for EDCs.

The mean values reported in Table 2 were statistically calculated handling left-censored observations by neglecting non-detectable values with the awareness that this decision can overestimate the values. In any case, all calculated EDI showed values under the regulatory limits currently available, except for BPA. In fact, EDI value for this bisphenol has been calculated also using two different substitution methods for the results reported to be below the LOD, when the non-detectable value was imputed as LOD (upper bound) and as zero (lower bound). Anyhow, the three obtained EDI values for BPA were above 0.2 ng/Kg/b.w./day, the limit established as tolerable daily intake (EDI) by EFSA in April 2023 [32]. It should be noted that this EFSA recommendation is recent, and before this period, the reference value was 4 μg/kg body weight(b.w.)/day and our calculated EDI values are well within this parameter.

Special attention needs to be directed towards PrPB (before classified as additive E216) used as an antimicrobial preservative in veterinary medicinal products. PrPB and its sodium salt, if allowed as an antimicrobial preservative in the intensive livestock farms, can pass into the animal’s milk and therefore be the route of PrPB transmission to the child. In 2015, the European Medicines Agency (EMA) suggested the ADI values reported in Table 2 [74].

Usually, phthalates monoesters are used as urinary markers of phthalate diesters, i.e., high concentrations of MEHP correlate with exposure to the parent chemical DEHP [87]. In our particular case, the presence of the monoesters phthalates in IF cannot be used to estimate EDI of phthalate diesters because the relationship between the diester intake and the amount of monoesters delivered into the milk is unknown. In any case, as all the IF herein tested were based on cows’ milk, this can be a possible via for phthalate monoesters pollution. In fact, phthalates and their metabolites can be incorporated to IF and transferred to the nursing child from consumer milk [88].

## 3. Materials and Methods

### 3.1. Reagents and Chemicals

A selection of 4 bisphenols [bisphenol A (BPA), bisphenol S (BPS), bisphenol F (BPF), and bisphenol F diglycidyl ether (BPFDGE)]; 7 parabens [Methylparaben (MePB), Ethylparaben (EtPB), n-Propylparaben (PrPB), iso-Propylparaben (iPrPB), n-Butylparaben (BuPB), and iso-Butylparaben (iBuPB)]; 14 diesters phthalates with its metabolites, mono-ester phthalates [Dimethyl phthalate (DMF), Diethyl phthalate (DEP), Dibutyl phthalate (DBP), Butylbenzyl phthalate (BBP), Di(2-ethylexyl) phthalate (DEHP), Di-n-octyl phthalate (DNOP), Monomethyl phthalate (MMP), monoethyl phthalate (MEP), Mono-n-butyl phthalate (MBP), Monobenzyl phthalate (MBzP), Mono(2-ethylexyl) phthalate (MEHP), Mono(2-ethyl-5-hydroxyhexyl) phthalate (MEHHP), Mono(2-ethyl-5-oxohexyl) phthalate (MEOHP), and Monooctyl phthalate (MnOP)]; 11 polycyclic aromatic hydrocarbons (PAHs) [Anthracene (ANTHR), Pyrene (Pyr), Phenanthrene (PHEN), Chrysene (CHRY), Benz[a]antracene (BAA), Benzo[b]fluoranthene (BBF), benzo[k]fluoranthene (BKF), Benzo[a]pyrene (BAP), Benzo[ghi]perylene (BGHIP), Dibenz[a,h]anthracene (DAA), and Indeno[1,2,3-cd]pyrene (IND)]; 3 polar pesticides [Glyphosate (GLY), Glufosinate (GLUF), and (Aminomethyl)phosphonic acid (AMPA)]; 2 pyrethroids [Cypermethrin (CP) and Cyfluthrin (CYFL)], and Chlorpyrifos (CPS), a solution standard with 16 metals [aluminum, arsenic, barium, bismuth, cadmium, cobalt, chromium, copper, manganese, molybdenum, nickel, lead, titanium, thallium, vanadium, zinc] were obtained from MERCK-Sigma Aldrich (Milano, Italy). Native PFAS precision and recovery standard solution with 27 PFAS [Perfluoro-n-butanoic acid (PFBA), Perfluoro-n-pentanoic acid (PFPeA), Perfluoro-n-hexanoic acid (PFHxA), Perfluoro-n-heptanoic acid (PFPHpA), Perfluoro-n-octanoic acid (PFOA), Perfluoro-n-nonanoic acid (PFNA), Perfluoro-n-decanoic acid (PFDA), Perfluoro-n-undecanoic acid (PFUnDA), Perfluoro-n-dodecanoic acid (PFDoDa),Perfluoro-n-tridecanoic acid (PFTrDA), Perfluoro-n-tetradecanoic acid (PFTrDA), Perfluoro-1 butanesulphonamide (PFBS), Perfluoropentanesulphonic acid (PFPeS), Perfluorohexanesulphonic acid (PFHxS), Perfluoroheptanesulphonic acid (PFHpS), Perfluorooctanesulphonic acid (PFOS), Perfluorononanesulfonic acid (PFNS), Perfluorododecanenesulfonic acid (PFDS), 2,3,3,3-tetrafluoro-2-(heptafluoropropoxy)propanoic acid (GenX), 4,8-Dioxa-3H-perfluorononanoic acid (ADONA), and Perfluoro(2-((6-chlorohexyl)oxy)ethanesulfonic acid) (9Cl-PF3ONS), 11-chloroeicosafluoro-3-oxaundecane-1-sulfonic acid (11Cl-PF3OUdS), 3,3,4,4,5,5,6,6,6-nonafluorohexane-1-sulfonic acid (4:2 FTS), 3,3,4,4,5,5,6,6,7,7,8,8,8-tridecafluorooctane-1-sulfonic acid (6:2 FTS), 3,3,4,4,5,5,6,6,7,7,8,8,9,9,10,10,10-heptadecafluorodecane-1-sulfonic acid (8:2 FTS), Perfluorobutane sulfonamide (FBSA), Perfluorooctane sulfonamide (FOSA)] was from Waters S.p.A. (Milano, Italy). Metals standard at 100 ppm was provided by CPAChem (Bogomilovo, Bulgary) and is a certified (CRM) NIST-traceable material.

All standards were selected and are summarized in Table S1.

Ultra-pure water was produced with a Milli-Q system (Sartorius Arium 611 VF, Varedo, Italy). The solvents acetonitrile (ACN), methanol, and formic acid (UPLC-MS grade) were supplied by Carlo Erba (Milano, Italy). QuEChERS (quick easy cheap effective rugged safe) extract pouches-EN method (salt packet containing 4 g MgSO_4_, 1 g NaCl), for QuEChERS extraction and QuEChERS fatty dispersive-SPE AOAC kit, 15 mL polypropylene tube containing 400 mg PSA, 400 mg C18EC, and 1200 mg MgSO_4_ were obtained from DTO Services Srl (Venezia, Italy). Strata-X^®^ SPE plate polimeric reverse phase (30 mg/well, 96-well plates, 33 μM Phenomenex, Castelmaggiore, Italy) beta-Glucuronidase from *E. Coli* K 12 was obtained from Merck (Milano, Italy). Vacuum extraction plate manifold for Oasis 96-well plates (Waters, Acquity, Milford, MA, USA). LC passivation solution containing 10 M medronic acid (1760 µg/mL, Methanol/Water (50:50)) was from Restek Corp. (Bellefonte, PA, USA).

### 3.2. Preparation of Standard Solutions

Each stock solution (1 μg/mL) containing a specific EDC group (4 different bisphenols, 11 polycyclic aromatic hydrocarbons, 14 phthalates, 7 parabens, 3 pesticides polar (glyphosate and its metabolites), and 3 pyrethroids and chlorpyrifos, respectively) was prepared by dissolving a weighed amount of substance or by dilution of a commercial standard solution in ACN and stored at −20 °C. Standard solutions (ng/mL) were prepared by dilution of the respective stock solutions with water–ACN (1:1, *v*/*v*).

### 3.3. Sampling Collection

Five commercial baby bottles selected among the most popular conventional brands available in the market with their teats were bought from the local pharmacies in Florence (Italy). Before use, sterilization of the baby bottles was performed following the package leaflet instruction, bottles were boiled in water for 5 min. In addition, one baby bottle with the daily used teat was provided by one mother, and 8 new feeding bottles were shipped directly from the neonatal Intensive Care Units of Parma and Reggio Emilia, and one kit for breast pumping was also collected and evaluated. Food-grade polypropylene (PP) was the plastic material used for the feeding bottles and for breast pumping, but one bottle material was polyethylene. Silicone is the material used for the teats. In this study, only two teats were made from latex rubber, and the rest were silicone. Moreover, all baby bottles presented the BPA-free label.

In order to evaluate if the baby bottles released any of the selected EDCs, we carried out experiments to simulate the contact between baby bottles and water. For this purpose, all baby and feeding bottles were filled with Milli-Q water that was left in contact for at least 48 h, and then collected for the analyses. These solutions were evaluated also after being flowed through their corresponding teats.

When using a pump, breast milk is usually collected by means of a “catheter” connected to the pump that delivers breast milk directly into the baby bottle. Milli-Q water was flowed over the catheter and further stored in the corresponding baby bottle at −20 °C until the analyses were performed.

Twenty different samples of IF were purchased. These were all included in the Italian National Register and commercialized in the pharmacies of Parma and Reggio Emilia, and were from the most widely used brands that were selected from the questionnaires filled in by the mothers enrolled in the Life MILCH project.

Selected samples included the following: 9 powder IFs type 1 used from birth up to 6 months; 8 powder IFs type 2 used after 6 months; 3 liquid IFs (2 IFs type 1 and 1 IF type 2).

Liquid formulas were packed in Tetrapack or high-density polyethylene (HDPE), whereas milk powders were stored in Al, Poliethylene terephthalate (PET), and Poliethylene (PE) containers.

Each sample of IF was prepared freshly before analysis following the instructions reported in the IF container. The powder sample was accurately weighed (4.5 g) using an analytical balance (Fisherbrand™ Bilance 220 g, Fisher Scientific Italia, Rodano, Milano, Italy) and was reconstituted with Milli-Q boiled water (30 mL, ~37 °C) in a polyethylene tube. A simplified solid-phase extraction (SPE) or dispersive SPE procedure was employed to extract all EDCs possibly present in the IF.

### 3.4. Instrumentation

#### 3.4.1. General Procedure UPLC-MS/MS Analysis

Ultra performance liquid chromatography UPLC (Waters, Acquity, Midfold, MA, USA) coupled to a Waters XEVO TQ-S triple quadrupole using electrospray ionization instrumentation was employed. The instrument was equipped with a Raptor biphenyl column (1.8 μm, 2.1 mm × 100 mm, Restek Srl, Milano, Italy) for phthalates, bisphenols, parabens, PAHs, and pyrethroids analysis, with a Raptor Polar X (2.7 μm 30 × 2.1 mm, Restek Srl, Milano, Italy) for glyphosate and its metabolites and with a ACQUITY UPLC^®^ HSS T3 (1.8 µm, 2.1 mm × 100 mm, Waters, Acquity, Midfold, MA, USA). In any case, the Ultrashield UPLC pre-column filter 0.2 μm frit was inserted. Column temperatures and flow rates are reported for each analyte in Table S2. Injection volumes were 10 μL. Used solvent systems and gradients are reported for each analyzed compound. All reagents were of at least UPLC reagent grade. Calibration curve ranges for each group of EDCs are reported in the Supporting Information (Tables S3–S9).

MS/MS parameters included bisphenols, parabens, glyphosate and its metabolites, and monoesters phthalates were analyzed in negative electrospray ionization while polycyclic aromatic hydrocarbons (PAHs), phthalate diesters, and pyrethroids and chlorpyrifos were analyzed in positive electrospray ionization.

For compounds, two MRM transitions were acquired for quantification and confirmation purposes. By direct infusion of standard solution (500 ng/mL), MRM data were optimized. The details of each MRM transition used for the MS/MS analysis detection are reported in the Supporting Information (Tables S10–S16). Data were acquired and processed using MassLynx™ software version 4.2 (Waters, Midfold, MA, USA) including the TargetLynx XS software version 4.2.

For the preparation of standard solutions, the stock solution containing the various analytes were prepared at 1 µg/mL in ACN; ten working standard solutions (0.05–2000 pg/mL for PFAS group and 0.01–500 ng/mL for the rest of compounds) were prepared for calibration curve plotting by diluting from stock solution with H_2_O/ACN (1:1) and the addition of 2 mM of ammonium acetate for PFAS.

#### 3.4.2. ICP-AES Analysis

An amount of approximately 0.5 g of IF was accurately weighted in PFA vessels and digested using an acidic solution of 2 mL supra pure HNO_3_ (obtained by sub-boiling distillation) and 0.5 mL of supra pure HCl (30%). The sample digestion was carried out by using a microwave digester (CEM Mars Xpress, CEM Corporation, Matthews, NC, USA) with a protocol including an initial 10 min ramp to 170 °C followed by a 20 min hold and a 40 min cool down. After the digestion, the samples were transferred to 25 mL vials and were diluted to ca. 10 mL with ultrapure water (UHQ—resistivity 18 MΩ cm—Milli-Q system by Millipore, Billerica, MA, USA) before analysis. The determination of heavy metal concentrations in the samples was performed in triplicate by a Varian 720-ES axial inductively coupled plasma atomic emission spectrometer (ICP-AES) (© Agilent Technologies, Inc., Santa Clara, CA, USA); 5 mL of each sample was spiked with 1.0 ppm of Ge used as an internal standard prior the analysis. The introduction system consisted of a concentric pneumatic nebulizer and a cyclonic spray chamber. Calibration standards were prepared by gravimetric serial dilution from commercial stock standard solution at 100 mg/L. The operating conditions were optimized to obtain maximum signal intensity, and between each sample, a rinse solution of 2% *v/v* HNO_3_ was used. All details are reported in Table S17.

### 3.5. Analytical Procedures

Stock standard solutions were prepared in ACN: H_2_O (50:50, *v*/*v*) or ACN: H_2_O (1:1) containing 2 mM ammonium acetate at a concentration of 1 ug/mL and stored at −20 °C. Ten standard solutions (ranging from 0.01 to 500 ng/mL or from 0.05 to 2000 pg/mL for PFAS) were prepared for all the compounds and used to obtain a calibration curve plotting. Curves with correlation coefficients (R^2^) ≥ 0.999 were generated. One blank and two control samples were included in each batch of samples. Calibration curves were obtained by spiking the standard mixture in the blank at low (10 ng/mL) and high concentrations (100 ng/mL). The quality control (QC) was accomplished following the same extraction and analysis procedures used for both real samples and standard solutions. Calibration curve plots for all target compounds (Table S1) with correlation coefficients (R^2^ > 0.99) were generated with 10 serial dilutions of the stock solutions. The intra-assay coefficients of variability (CV) were ≤10% and inter-assay CV were ≤12% for all analytes at the two QC levels. For the 7 UPLC-MS/MS methods set up for each group of assessed or suspected EDCs (Table S2), the accuracy of the method (QA) was calculated in terms of relative recovery estimated by spiking the pool of blank formula milk at a fixed concentration. A recovery percentage within the range 100% ± 20% was considered acceptable. Inter- and intra-day precision was also evaluated by measuring five replicates of each sample daily and at least for five consecutive days, respectively.

The limit of quantification (LOQ) for each analyte was determined by analyzing ten different standard concentrations with progressively lower concentrations. The LOQ was set where the imprecision was CV ≤ 20% and the signal to noise ratio was >10 on all 6 days. For each analyte, the limit of detection (LOD) was set using a signal-to-noise ratio ≥ 3 on all 6 days.

Intra-assay imprecision was determined by analyzing two urine pools on the same day in 10 replicates. Inter-assay imprecision was assessed by measuring two urine pools on 10 different days.

### 3.6. Formula Milk Samples Pre-Treatment

#### 3.6.1. QuEChERS for Phthalates, Bisphenols, PAHs, Parabens, and Pyrethroid Extractions

A simplified QuEChERS procedure was employed to extract chemicals from IF. Each IF (30 mL) was prepared according to the indications given in the feeding table. In polypropylene tubes for the hydrolysis of conjugated species, the β-glucuronidase solution (1 mL,) was added to IF (10 mL). The enzymatic solution was prepared by dissolving the enzyme-purified powder in ammonium acetate 1 M (pH = 5) to obtain a solution of 3500 U/mL. The mixture was incubated overnight at 37 °C in a C24 incubator shaker (New Brunswick Scientific, Edison, NJ, USA) to finalize the deconjugation of samples. ACN (10 mL) was added to the mixture, and the solution was shaken for 1 min using a vortex and then put in an ice bath. QuEChERS salts (4 g anhydrous MgSO_4_, 1 g NaCl) were added to the mixture, vigorously vortexed, and replaced in the ice bath. The falcon with salts and milk was centrifuged at 4000 rpm at 4 °C for 20 min (Heraeus^®^ Megafuge^®^, Kendro Laboratory Products GmbH., Hanau, Germany). The supernatant was transferred into a 15 mL QuEChERS fatty dispersive-SPE polypropylene tube (Agilent dispersive SPE, 15 mL, Association of Official Analytical Chemists (AOAC) method, ©Agilent Technologies, Inc, Santa Clara, CA, USA). The mixture was shaken for 1 min and centrifuged for 15 min at 4000 rpm at 4 °C. The cleaned supernatant was transferred into a glass tube (15 mL) and evaporated to dryness. The dry residue was then dissolved in 300 μL of ACN: water (50:50, *v*/*v*) and placed into an eppendorf and ultra-centrifuged. The final supernatant was transferred into an injection vial and analyzed on the UPLC–MS/MS system.

#### 3.6.2. Milk Sample Filtration for Glyphosate and Its Metabolite Extractions

Each IF was prepared according to the indications given in the feeding table. The β-glucuronidase solution (1 mL) was added to the IF (1 mL) in polypropylene tubes for the hydrolysis of the conjugated species, and the suspension was incubated overnight at 37 °C in a C24 incubator shaker (New Brunswick Scientific, Edison, NJ, USA). ACN (2 mL), the mixture was shaken for 1 min with a vortex and put in an ice bath to precipitate the proteins. In the meantime, the STRATA-X^®^ SPE plate (33 μm, 30 mg/well, 96-well plates, Phenomenex, Torrance, CA, USA) was preconditioned with 2 mL ACN: water (50:50, *v*/*v*). After centrifugation (20 min, 4000 rpm at 4 °C, 30 min), the aqueous layer was filtered through to SPE column Strata-X^®^ SPE clean 96-well plate using a vacuum extraction plate manifold for Oasis 96-well plates (Waters, Acquity, Milford, MA, USA). The plate was then dried under vacuum for 1 min and each eluate collected into the clean 96-well plate for analysis by LC-MS/MS.

#### 3.6.3. QuEChERS for PFAS

The full QuEChERS extraction and dSPE cleanup for PFAS compounds is fully outlined in the described protocol [58]. Briefly, 5 mL of pure water were added to 10 mL of IF. Then, 10 mL of acetonitrile and 150 uL of formic acid were added and shaken for 1 min. Then, AOAC QuEChERs salts (6 g MgSO_4_ and 1.5 g of sodium acetate) were added and mixed for 5 min and then centrifuged for 5 min at 4000 rpm. A total of 5 mL of the supernatant were transferred to a 15 mL dSPE (1200 mg MgSO_4_, 400 mg PSA, and 400 mg C18), shaken for 1 min, and centrifugated for 5 min at 4000 rpm. Solution was diluted 1:1 in 2 mM ammonium acetate.

### 3.7. Overall Dietary Exposure to Single EDCs in the Infants

EDCs quantification in IF samples and in the baby bottles were used to evaluate the overall dietary exposure of infants by calculating the estimated dietary intake (EDI). The concentrations of EDCs which were found equal to or greater than their respective LOQ in the IF were considered for estimating the daily intake. EDI was calculated combining the EDCs mean concentrations found in samples with the corresponding average milk consumption at the arbitrary age of 1 month.

The 50th percentile average intakes, respectively, for formula milk, calculated for the age of 1 month were calculated as follows:

EDI (μg kg^−1^ b.w. per day) = EDCs mean Conc × IF/b.w.

where EDCs Conc (μg g^−1^) is the mean concentration of each EDC in the sample, IF is the daily IF consumption per day (g) at 1 month of age (93 g dry weight/day, 50th percentile), and b.w. is average body weight at 1 month (4.25 kg, 50th percentile) in accordance with the Food and Agriculture Organization of the United Nations/World Health Organization (FAO/WHO) and with the help of weight growth charts by WHO. [71] EDI values were calculated using the means of the EDCs in the different type 1 formulas (N = 11).

## 4. Conclusions

With the idea in mind to evidence potential risks in infant diet associated with contamination of formula milk by EDCs, in the present study, 85 chemical compounds defined as EDCs or suspected EDCs were quantified in different infant formula milks and containers.

The release of EDCs in five commercial baby bottles with their teats, in eight feeding bottles used in the neonatal intensive care units, and one kit of a manual breast pump was lower compared to IFs. This is probably due to the normative restrictions proposed by the Scientific Committee for Food applied to plastic food contact materials (Commission Regulation (EU) 2006/141 and subsequent amendments; in Italy, it is regulated by Ministerial Decree (DM) no. 82 of 9 April 2009 and subsequent modifications), especially for childcare. Moreover, the present migration study also suggested that the repeated use of the baby bottles did not increase the leaking of EDCs.

In IF, at variance, bisphenols, parabens, phthalates, and PFAS were largely present. Among PAHs, IND was found in one sample only.

Based on the data reported herein, particular caution is recommended during the inspection of the investigated formulas by public health authorities regarding contamination by toxic metals, especially Al, consistently identified in most IFs.

It is noteworthy to mention some limitations for this study. In particular, difficult access to the possible sources of contamination, the whole raw material (milk of animal origin), the manufacturing processes to obtain powdered milk, and packaging have to be considered. For example, possible release of phthalates from PVC tubes used for milking in big farms or bags containing animal feed can be unpredictable sources of contamination that are not easy to control. Moreover, contamination could be closely linked to urban or rural environmental pollutants interfering with the infant formula production process, particularly the volatile properties of some EDCs, such as PAHs.

Finally, the chemical risk assessment was traditionally conducted on a chemical-by-chemical basis, thus, neglecting possible combined effects. Focusing only on food exposure and not on other possible contaminants, newborns are also exposed to many different chemical substances that can have the same toxicological effect. Herein, we describe the child exposure to different chemical compounds found in formula milk, although individually low exposure is observed for each substance, we cannot overlook the combined effects of the substances found. The presence of EDCs in amounts below those reported for the legal limits does not exclude effects from their interaction in mixtures. Many of these compounds are assessed or suspected of impacting human health, and their combination could exacerbate their harmful effects. In this context, the EU commission indicates that the exposure analysis should be localized depending on the modes of action. If chemicals have similar modes of action, there is a potential for cumulative effects when such chemicals are present together in a mixture (even when the concentration of each substance is below its “safe level”) and then, the concentration/dose addition approach is preferred in order to assure an adequate level of protection. On the other hand, in the case of chemicals with independent modes of action, the establishment of “safe levels” based on the assessment of individual substances appears, in relation to human health, to provide a sufficient safeguard against possible negative effects from mixtures/combinations [89]. Methodologies herein described will be further applied to breast milk, but also urines and sera, in the context of the LIFE EU project MILCH.

## Figures and Tables

**Table 1:** Assessed and suspected EDCs concentrations in ng/g dry weight ^a^ of infant formula (N = 20).

EDC Group	Compound	Frequency (%)	Mean ± SD	Median	Min–Max
Bisphenol	BPA	3/20 (15)	1.76 ± 1.28	2.25	0.31–2.73
	BPFDGE	4/20 (20)	5.43 ± 1.97	6.35	2.48–6.54
Parabens	MePB	3/20 (15)	2.9 ± 2.95	1.36	1.04–6.29
	EtPB	1/20 (5)	0.32	0.32	
	PrPB	1/20 (5)	0.12	0.12	
Polycyclic aromatic hydrocarbons (PAHs)	IND	1/20 (5)	0.32	0.32	0.32–0.32
Phthalates	DMP	9/20 (45)	0.37 ± 0.19	0.34	0.11–0.75
	MMP	6/20 (30)	0.07 ± 0.11	0.03	0.02–0.29
	DEP	17/20 (85)	1.51 ± 1.56	1.27	0.28–7.21
	MEP	2/20 (10)	0.03 ± 0.03	0.03	0.01–0.05
	MBP	14/20 (70)	0.48 ± 0.93	0.22	0.04–3.65
	BBzP	20/20 (100)	1.27 ± 0.91	0.94	0.68–3.45
	DEHP	8/20 (40)	1.09 ± 0.76	0.89	0.38–2.79
	MEHP	14/20 (70)	0.40 ± 0.61	0.21	0.09–2.40
	DNOP	10/20 (50)	0.68 ± 0.40	0.60	0.23–1.52
	MnOP	14/20 (70)	0.24 ± 0.35	0.12	0.05–1.46
Perfluoroalkyl substances (PFASs)	PFOA	4/20 (20)	0.02 ± 0.01	0.03	0.01–0.03
	PFBA	1/20 (5)	0.09	0.09	
	PFPHpA	1/20 (5)	0.02	0.02	
	PFDA	5/20 (25)	0.01 ± 0.001	0.01	0.009–0.011
	PFBS	18/20 (90)	0.096 ± 0.03	0.09	0.04–0.13
Metals	Al	20/20 (100)	546 ± 254	528	192–1005
	As	4/20 (20)	11 ± 3.7	11	7.3–16.3
	Ba	20/20 (100)	178 ± 101.8	156.5	60.6–458.2
	Cd	19/20 (95)	3 ± 2.1	2	0.4–7.7
	Co	20/20 (100)	13 ± 5.9	10.8	6.5–24.7
	Cr	20/20 (100)	81 ± 47.3	71.1	28–252
	Cu	20/20 (100)	4168 ± 1172	3953	3085–8649
	Mn	20/20 (100)	768 ± 538	571	173–1820
	Mo	20/20 (100)	116 ± 72.1	100.8	21.3–269.3
	Ni	20/20 (100)	32 ± 15.1	30.6	13.1–65.9
	Ti	20/20 (100)	35 ± 31.8	25.8	5.8–104.0
	V	4/20 (20)	13 ± 13.3	6.9	6–33.3
	Zn	20/20 (100)	34,740 ± 6942.4	32,042.7	25,883.2–52,680.8

^a^ Mean ± SD, median and range.

**Table 2:** Estimated daily intake (EDI, ng/Kg/b.w./day) for each compound (y) in infant formula type 1, using the WHO curves to estimate mean weight at days of life. Tolerable daily intake (TDI, ng/Kg/b.w./day) is according to EFSA.

EDCs	Chemical	Frequency (%)	Mean ± SD	Median	Min–Max	EDI for Infant’s Weight (kg) at the 50th Percentile (Average for Age)	TDI Values Reported in the Literature
Bisphenols	BPA	1/11 (9.1)	2.73 ^a^	2.73	2.73–2.73	59.74	0.2 (ng/kg/b.w./day) [32]
			0.3 ± 0.86 ^b^	0.02	0.49–59.74	6.46	
			0.25 ± 0.82 ^c^	0	0–59.74	5.43	
	BPFDGE	1/11 (9.1)	6.39	6.39	6.39–6.39	139.84	Maximum PDI of BPFDGE < 3.4 µg/kg body weight/day [72]
Parabens	MePB	1/11 (9.1)	1.04	1.04	1.04–1.04	22.7	Σ of parabens in food for EFSA 0–10 mg/kg b.w. [34]
	PrPB	1/11 (9.1)	0.12	0.12	0.12–0.12	2.62	ADI for PrPB at 1.25 mg/kg/b.w./day) [73,74,75].
Phthalates	DMP	7/11 (64)	0.41 ± 0.43	0.36	0.11–0.75	8.92	0.9–7.2 and 1.6–11.7 μg/kg b.w. for DBP, BBP, 50 μg/kg b.w. per day for DEHP [76]
	MMP	5/11 (45)	0.08 ± 0.09	0.03	0.02–0.29	1.73	
	DEP	9/11 (82)	1.91 ± 2.02	1.27	0.8–7.21	42.23	
	MEP	2/11 (18)	0.03 ± 0.02	0.03	0.01–0.05	1.11	
	MBP	9/11 (82)	0.57 ± 1.15	0.22	0.04–3.65	12.64	
	BBzP	11/11 (100)	1.09 ± 0.46	0.91	0.68–2.32	24.14	
	DEHP	5/11 (45)	1.15 ± 0.89	0.71	0.38–2.79	25.54	
	MEHP	9/11 (82)	0.50 ± 0.74	0.9	0.09–2.44	11.16	
	DNOP	6/11 (62)	0.67 ± 0.4	0.6	0.34–1.12	14.81	
	MnOP	9/11 (82)	0.3 ± 0.45	0.12	0.05–1.46	6.65	
PFASs	PFOA	2/11 (18)	0.07		0.03–0.11	0.3	
	PFPHpA	1/11 (9.1)	0.102	0.102	0.102	0.02	TWI: Σ of PFAS for EFSA 4.4 ng/kg body weight/week [49]
	PFDA	2/11 (18)	0.05		0.04–0.06	0.01	
	PFBS	11/11 (100)	0.46		0.17–0.74	0.07	
Metals	Al	11/11 (100)	493 ± 252	390	193–851	10,778	PTWI of 7 mg/kg for FAO Expert Committee [77] Sub-chronic oral reference dose of 1 mg/kg per day for ATSDR [78,79]
	As	2/11 (18)	10 ± 3	10	7–12	209	PTWI of 0.015 mg/kg of body weight Sub-chronic oral reference dose of 0.0003 mg/kg per day for [79]
	Ba	11/11 (100)	151 ± 99	133	61–405	3315	
	Cd	10/11 (91)	2 ± 2	1	1–8	52	PTWI of 2.5 μg kg^−1^ b.w. by [80] Sub-chronic oral reference dose of 0.001 mg/kg per day by [79]
	Co	11/11 (100)	15 ± 7	14	7–25	320	
	Cr	11/11 (100)	64 ± 26	59	28–131	1399	300 mg/kg b.w. PTDI [81]
	Cu	11/11 (100)	4510 ± 1490	4006	3319–8649	98,679	0.20 mg/day for infant 0–6 months old [82]
	Mn	11/11 (100)	741 ± 561	597	185–1774	16,212	0.0504 and 5.04 μg g^−1^ in formula as minimum and maximum content [83] upon [84]
	Mo	11/11 (100)	95 ± 68	86	21–244	2072	
	Ni	11/11 (100)	32 ± 15	31	13–65	707	2.8 mg/kg b.w./day PTDI [85]
	Ti	11/11 (100)	31 ± 32	22	6–104	686	
	V	2/11 (18)	7 ± 1	7	6–7	152	
	Zn	11/11 (100)	35,252 ± 8504	31,194	25,883–52,681	771,407	a tolerable upper limit of 7 mg/day for SCF [86]

Mean values of BPA calculated with the following statistical methods to handle left-censored observations: ^a^ neglecting non-detected value; using substitution method for results reported to be below the LOD, when the non-detected value was imputed: ^b^ LOD (upper bound), ^c^ as zero (lower bound). Abbreviations: ADI—acceptable daily intake; ATSDR—Agency for Toxic Substances and Disease Registry; PDI—probable daily intakes; PTDI—provisional tolerable daily intake; PTWI—provisional tolerable weekly intake; SCF—scientific committee on food; TWI—tolerable weekly intake.

## Data Availability

Data are contained within the article and in the Supplementary Materials Section.

## Supplementary Materials

The following supporting information can be downloaded at: https://www.mdpi.com/article/10.3390/molecules29225434/s1. Table S1. Assessed or suspected endocrine disrupting chemicals selected for the study. Table S2. UPLC–MS/MS analysis. Table S3. Linearity and sensitivity for each Bisphenol with the proposed analytical method. Table S4. Linearity and sensitivity for each Paraben with the proposed analytical method. Table S5. Linearity and sensitivity for each PAH with the proposed analytical method. Table S6. Linearity and sensitivity for each pesticide with the proposed analytical method. Table S7. Linearity and sensitivity for each Phthalate with the proposed analytical method. Table S8. Linearity and sensitivity for each Pyrethroid with the proposed analytical method. Table S9. Linearity and sensitivity for each PFAS with the proposed analytical method. Table S10. Analytical Rt and MRM transition parameters of bisphenols. Table S11. Analytical Rt and MRM transition parameters of Parabens. Table S12. Analytical Rt and MRM transition parameters of PAHs. Table S13. Analytical Rt and MRM transition parameters of glyphosate and its metabolites. Table S14. Analytical Rt and MRM transition parameters for phathalates. Table S15. Analytical Rt and MRM transition parameters for pyrethroids and chlorpyrifos; Table S16. Analytical Rt and MRM transition parameters for PFAS; Table S17. Linearity and sensitivity for each metal and metalloid measured by ICP-AES.
