# Supplementary material for: Study of Endocrine-Disrupting Chemicals in Infant Formulas and Baby Bottles: Data from the European LIFE-MILCH PROJECT
Source: Molecules. 2024 Nov 18;29(22):5434. doi: 10.3390/molecules29225434 (PMC11597460; doi:10.3390/molecules29225434)
Supplement: Supplementary file 1 [file molecules-29-05434-s001.zip › molecules-3275222-supplementary.pdf]

Supplementary materials

Study of Endocrine-Disrupting Chemicals in Infant Formulas and Baby Bottles: Data from the European LIFE-MILCH PROJECT

Francesca Nuti [1,2,†], Feliciana Real Fernández [1,3,†], Mirko Severi [2], Rita Traversi [2], Vassilios Fanos [4], Maria Elisabeth Street [5,6], Paola Palanza [5,7], Paolo Rovero [1,8], Anna Maria Papini [1,2,*]

[1] Interdepartmental Research Unit of Peptide and Protein Chemistry and Biology (Peptlab) and Centre of Competences in Molecular Diagnostics and Life Sciences (MoD&LS), University of Florence, 50019 Sesto Fiorentino, Italy; francesca.nuti@unifi.it (F.N.); feliciana.realfernandez@iccom.cnr.it (F.R.F.); paolo.rovero@unifi.it (P.R.)
[2] Department of Chemistry "Ugo Schiff", University of Florence, 50019 Sesto Fiorentino, Italy; mirko.severi@unifi.it (M.S.); rita.traversi@unifi.it (R.T.)
[3] Institute of Chemistry of Organometallic Compounds, National Research Council (ICCOM-CNR), 50019 Sesto Fiorentino, Italy
[4] Section of Neonatal Intensive Care Unit, Department of Paediatrics, Puericulture Institute and Neonatal Section, Azienda Mista and University of Cagliari, 09124 Cagliari, Italy; vafanos@tiscali.it (V.F.)
[5] Department of Medicine and Surgery, University of Parma, 43126 Parma, Italy; mariaelisabeth.street@unipr.it (M.E.S.)
[6] Unit of Pediatrics, University Hospital of Parma, 43126 Parma, Italy
[7] Behavioral Biology Laboratory, University of Parma, 43124 Parma, Italy
[8] Department of Neurosciences, Psychology, Drug Research and Child Health (NeuroFarBa), University of Florence, 50019 Sesto Fiorentino, Italy
* Correspondence: annamaria.papini@unifi.it
† These authors contributed equally to this work.

Abbreviations: LOD: limit of detection; LOQ limit of quantification; MRM multiple reaction monitoring; UPLC Ultra performance liquid chromatography; ICP-AES Inductively coupled plasma atomic emission spectroscopy; MS mass spectrometry; ACN acetonitrile; ESI electrospray ionization; IF infant formula; Rt retention time.

General procedure

UPLC-MS/MS analysis

Ultra performance liquid chromatography UPLC (Waters, Acquity, Midfold, MA, USA) coupled to a Waters XEVO TQ-S triple quadrupole using electrospray ionization instrumentation was employed. The instrument was equipped with a Raptor biphenyl column (1.8 µm, 2.1 mm x 100 mm, Restek Srl, Milano, Italy) for phthalates, bisphenols, parabens, PAHs, and pyrethroids analysis, with a Raptor Polar X (2.7um 30 x 2.1mm, Restek Srl, Mi-lano, Italy); for glyphosate and its metabolites with a ACQUITY UPLC® HSS T3 (1.8 µm, 2.1 mm x 100 mm, Waters, Acquity, Midfold, MA, USA). In any case, the Ultrashield UPLC pre-column filter 0,2µm frit was inserted. Column temperatures and flow rates are reported for each analyte in Table S2. Injection volumes: 10 µL. Used solvent systems and gradients are reported for each analysed compound. All reagents were of at least UPLC reagent grade. Calibration curve ranges for each group of EDCs are reported herein (Tables S3-S9).

MS/MS Parameters: Bisphenols, parabens, glyphosate and its metabolites, monoesters phthalates, and perfluoroalkyl substances (PFAS) were analysed in negative electrospray ionization while polycyclic aromatic hydrocarbons (PAHs), phthalate diesters, and pyrethroids and chlorpyrifos were analyzed in positive electrospray ionization.

For compounds, two MRM transitions were acquired for quantification and confirmation purposes. By direct infusion of standard solution (500 ng/mL) MRM data were optimized. The details of each MRM transition used for the MS/MS analysis detection are here in reported (Tables S10-S16). Data were acquired and processed using MassLynx™ software version 4.2 (Waters, Midfold, MA, USA) including the TargetLynx XS software.

ICP-AES analysis

An amount of approximately 0.5 g of IF was accurately weighted in PFA vessels and digested using an acidic solution of 2 mL suprapure HNO_3 (obtained by sub-boiling distillation) and 0.5 mL of suprapure HCl (30%). The sample digestion was carried out by using a microwave digester (CEM Mars Xpress, CEM Corporation, Matthews, NC; USA) with a protocol including an initial 10 min ramp to 170°C followed by a 20 min hold and a 40 min cool down. After the digestion, the samples were transferred to 25 mL vials and were diluted to ca. 10 mL with ultrapure water (UHQ – resistivity 18 MΩ cm – Milli-Q system by Millipore, Billerica, MA, USA) before analysis. The determination of heavy metal concentrations in the samples was performed in triplicate by a Varian 720-ES axial Inductively Coupled Plasma Atomic Emission Spectrometer (ICP-AES, Agilent Technologies, Inc, Santa Clara, CA, USA); 5 mL of each sample was spiked with 1.0

ppm of Ge used as an in-ternal standard prior the analysis. The introduction system consisted of a concentric pneumatic nebulizer and a cyclonic spray chamber. Calibration standards were prepared by gravimetric serial dilution from commercial stock standard solution at 100 mg/L. The operating conditions were optimized to obtain maximum signal intensity, and between each sample, a rinse solution of 2% v/v HNO_3 was used. All details are reported in Table S17.

Table S1. Assessed or suspected endocrine disrupting chemicals selected for the study			
**Group**	**Chemical compound**	**Abbreviations**	**CAS number**
**Bisphenols**	Bisphenol A<br>4,4'-(1-Methylethylidene)bisphenol	BPA	80-05-7
	Bisphenol S<br>4,4'-Sulfonylbisphenol	BPS	80-09-1
	Bisphenol F<br>4,4'-Methylenebisphenol	BPF	620-92-8
	Bisphenol F Bis(3-chloro-2-hydroxypropyl)ether<br>1-chloro-3-[4-[[4-(3-chloro-2-hydroxypropoxy)phenyl]methyl]phenoxy]propan-2-ol	BPFDGE	374772-79-9
**Parabens**	Methylparaben<br>Methyl 4-hydroxybenzoate	MePB	99-76-3
	Ethylparaben<br>Ethyl 4-hydroxybenzoate	EtPB	120-47-8
	n-Propylparaben<br>Propyl 4-hydroxybenzoate	PrPB	94-13-3
	iso-Propylparaben<br>Propan-2-yl 4-hydroxybenzoate	iPrPB	4191-73-5
	n-Butylparaben<br>Butyl 4-hydroxybenzoate	BuPB	94-26-8
	iso-Butylparaben<br>2-Methylpropyl 4-hydroxybenzoate	iBuPB	4247-02-3
	Benzylparaben<br>Propyl 4-hydroxybenzoate	BzPB	94-18-8
**Polycyclic aromatic hydrocarbons (PAHs)**	Anthracene<br>Anthracene	ANTHR	120-12-7
	Pyrene<br>Pyrene	PYR	129-00-0
	Phenanthrene<br>Phenanthrene	PHEN	85-02-8
	Chrysene<br>Chrysene	CHRY	218-02-9
	Benz[a]antracene<br>Tetraphene	BAA	56-55-3
	Benzo[b]fluoranthene<br>Benzo[e]acephenanthrylene	BBF	205-99-2
	Benzo[k]fluoranthene<br>benzo[k]fluoranthene	BKF	207-08-9
	Benzo[a]pyrene<br>Benzo[pqr]tetraphene	BAP	50-32-8
	Benzo[ghi]perylene<br>Benzo[ghi]perylene	BGHIP	191-24-2

	Dibenz[a,h]anthracene<br>Benzo[k]tetraphene	DAA	53-70-3
	Indeno[1,2,3-cd]pyrene<br>Indeno[1,2,3-cd]pyrene	IND	193-39-5
**Polar pesticides**	Glyphosate<br>2-(Phosphonomethylamino)acetic acid	GLY	1071-83-6
	Glufosinate<br>2-Amino-4-[hydroxy(methyl)phosphoryl]butanoic acid	GLUF	51276-47-2
	AMPA<br>(Aminomethyl)phosphonic acid	AMPA	1066-51-9
**Pyrethroids and chlorpyrifos**	Cypermethrin<br>[cyano-(3-phenoxyphenyl)methyl] 3-(2,2-dichloroethenyl)-2,2-dimethylcyclopropane-1-carboxylate	CP	52315-07-8
	Cyfluthrin<br>[cyano-(4-fluoro-3-phenoxyphenyl)methyl] 3-(2,2-dichloroethenyl)-2,2-dimethylcyclopropane-1-carboxylate	CYFL	68359-37-5
	Chlorpyrifos<br>Diethoxy-sulfanylidene-(3,5,6-trichloropyridin-2-yl)oxy-λ5-phosphane	CPS	2921-88-2
**Phthalates**	Dimethyl phthalate<br>Dimethyl benzene-1,2-dicarboxylate	DMP	131-11-3
	* Monomethyl phthalate<br>2-(Methoxycarbonyl)benzoic acid	MMP	4376-18-5
	Diethyl phthalate<br>Diethyl benzene-1,2-dicarboxylate	DEP	84-66-2
	* Monoethyl phthalate<br>2-(Ethoxycarbonyl)benzoic acid	MEP	2306-33-4
	Dibutyl phthalate<br>Dibutyl benzene-1,2-dicarboxylate	DBP	84-74-2
	# Mono-n-butyl phthalate<br>2-(Butoxycarbonyl)benzoic acid	MBP	131-70-4
	Butylbenzyl phthalate<br>2-O-benzyl 1-O-butyl benzene-1,2-dicarboxylate	BBP	85-68-7
	* Monobenzyl phthalate<br>2-[(Benzyloxy)carbonyl]benzoic acid	MBzP	2528-16-7
	Di(2-ethylexyl) phthalate<br>Di(2-ethylhexyl)benzene-1,2-dicarboxylate	DEHP	117-81-7
	* Mono(2-ethylexyl) phthalate<br>2-(2-Ethylhexoxycarbonyl)benzoic acid	MEHP	4376-20-9
	* Mono(2-ethyl-5-hydroxyhexyl) phthalate<br>2-(2-Ethyl-5-hydroxyhexoxycarbonyl)benzoic acid	MEHHP	40321-99-1
	* Mono(2-ethyl-5-oxohexyl) phthalate<br>2-(2-Ethyl-5-oxohexoxycarbonyl)benzoic acid	MEOHP	40321-98-0
	Di-n-octyl phthalate<br>Dioctyl benzene-1,2-dicarboxylate	DNOP	117-84-0
	* Monooctyl phthalate	MnOP	

	2-(Octoxycarbonyl)benzoic acid		5393-19-1
**Perfluoroalkyl substances (PFAS)**	2,2,3,3,4,4,4-heptafluorobutanoic acid<br>Perfluoro-n-butanoic acid	PFBA	375-22-4
	2,2,3,3,4,4,5,5,5-nonafluoropentanoic acid<br>Perfluoro-n-pentanoic acid	PFPeA	2706-90-3
	2,2,3,3,4,4,5,5,6,6,6-undecafluorohexanoic acid<br>Perfluoro-n-hexanoic acid	PFHxA	307-24-4
	2,2,3,3,4,4,5,5,6,6,7,7,7-tridecafluoroheptanoic acid<br>Perfluoro-n-heptanoic acid	PFPHpA	375-85-9
	2,2,3,3,4,4,5,5,6,6,7,7,8,8,8-pentadecafluorooctanoic acid<br>Perfluoro-n-octanoic acid	PFOA	335-67-1
	2,2,3,3,4,4,5,5,6,6,7,7,8,8,9,9,9-heptadecafluorononanoic acid<br>Perfluoro-n-nonanoic acid	PFNA	375-95-1
	2,2,3,3,4,4,5,5,6,6,7,7,8,8,9,9,10,10,10-nonadecafluorodecanoic acid<br>Perfluoro-n-decanoic acid	PFDA	335-76-2
	2,2,3,3,4,4,5,5,6,6,7,7,8,8,9,9,10,10,11,11,11-henicosafluoroundecanoic acid<br>Perfluoro-n-undecanoic acid	PFUnDA	2058-94-8
	2,2,3,3,4,4,5,5,6,6,7,7,8,8,9,9,10,10,11,11,12,12,12-tricosafluorododecanoic acid Perfluoro-n-dodecanoic acid	PFDoDA	307-55-1
	2,2,3,3,4,4,5,5,6,6,7,7,8,8,9,9,10,10,11,11,12,12,13,13,13-pentacosafluorotridecanoic acid<br>Perfluoro-n-tridecanoic acid	PFTrDA	72629-94-8
	2,2,3,3,4,4,5,5,6,6,7,7,8,8,9,9,10,10,11,11,12,12,13,13,14,14,14-heptacosafluorotetradecanoic acid<br>Perfluoro-n-tetradecanoic acid	PFTreDA	376-06-7
	1,1,2,2,3,3,4,4,4-nonafluorobutane-1-sulfonic acid<br>Perfluoro-1 butanesulphonamide	PFBS	375-73-5
	1,1,2,2,3,3,4,4,5,5,5-undecafluoropentane-1-sulfonic acid<br>Perfluoropentanesulphonic acid	PFPeS	2706-91-4
	1,1,2,2,3,3,4,4,5,5,6,6,6-tridecafluorohexane-1-sulfonic acid<br>Perfluorohexanesulphonic acid	PFHxS	355-46-4
	1,1,2,2,3,3,4,4,5,5,6,6,7,7,7-pentadecafluoroheptane-1-sulfonic acid<br>Perfluoroheptanesulphonic acid	PFHpS	375-92-8
	1,1,2,2,3,3,4,4,5,5,6,6,7,7,8,8,8-heptadecafluorooctane-1-sulfonic acid<br>Perfluorooctanesulphonic acid	PFOS	1763-23-1
	1,1,2,2,3,3,4,4,5,5,6,6,7,7,8,8,9,9,9-nonadecafluorononane-1-sulfonic acid<br>Perfluorononanesulfonic acid	PFNS	68259-
	1,1,2,2,3,3,4,4,5,5,6,6,7,7,8,8,9,9,10,10,11,11,12,12,12-pentacosafluorododecane-1-sulfonic acid<br>Perfluorododecanenesulfonic acid	PFDS	79780-39-5

	2,3,3,3-tetrafluoro-2-(1,1,2,2,3,3,3-heptafluoropropoxy)propanoic acid<br>2,3,3,3-tetrafluoro-2-(heptafluoropropoxy)propanoic acid	GenX (HFPO-DA)	13252-13-6
	2,2,3-trifluoro-3-[1,1,2,2,3,3-hexafluoro-3-(trifluoromethoxy)propoxy]propanoic acid<br>4,8-Dioxa-3H-perfluorononanoic acid	ADONA	919005-14-4
	2-(6-chloro-1,1,2,2,3,3,4,4,5,5,6,6-dodecafluorohexoxy)-1,1,2,2-tetrafluoroethanesulfonic acid<br>Perfluoro(2-((6-chlorohexyl)oxy)ethanesulfonic acid)	9Cl-PF3ONS	756426-58-1
	2-(8-chloro-1,1,2,2,3,3,4,4,5,5,6,6,7,7,8,8-hexadecafluorooctoxy)-1,1,2,2-tetrafluoroethanesulfonic acid<br>11-chloroeicosafluoro-3-oxaundecane-1-sulfonic acid	11Cl-PF3OUdS	763051-92-9
	1H,1H,2H,2H-Perfluorohexanesulphonic acid<br>3,3,4,4,5,5,6,6,6-nonafluorohexane-1-sulfonic acid	4 :2 FTS	757124-72-4
	1H,1H,2H,2H-perfluorooctanesulfonic acid<br>3,3,4,4,5,5,6,6,7,7,8,8,8-tridecafluorooctane-1-sulfonic acid	6:2 FTS	27619-97-2
	31H,1H,2H,2H-Perfluorodecanesulfonic acid<br>3,3,4,4,5,5,6,6,7,7,8,8,9,9,10,10,10-heptadecafluorodecane-1-sulfonic acid	8 :2 FTS	39108-34-4
	1,1,2,2,3,3,4,4,4-nonafluorobutane-1-sulfonamide<br>Perfluorobutane Sulfonamide	FBSA	30334-69-1
	Perfluorooctanesulfonamide<br>1,1,2,2,3,3,4,4,5,5,6,6,7,7,8,8,8-heptadecafluorooctane-1-sulfonamide	FOSA	754-91-6
**Metals**	Aluminum	Al	7429-90-5
	Arsenic	As	7440-38-2
	Barium	Ba	7440-39-3
	Bismuth	Bi	7440-69-9
	Cadmium	Cd	7440-43-9
	Cobalt	Co	7440-48-4
	Chromium	Cr	7440-47-3
	Copper	Cu	7440-50-8
	Manganese	Mn	7439-96-5
	Molybdenum	Mo	7439-98-7
	Nickel	Ni	7440-02-0

	Lead	Pb	7439-92-1
	Titanium	Ti	7440-32-6
	Thallium	Tl	7440-28-0
	Vanadium	V	7440-62-2
	Zinc	Zn	7440-66-6

Table S2. UPLC–MS/MS analysis						
**EDCs Groups**	**Column temp. (°C)**	**Flow rate (mL/min)**	**Solvent system**	**Gradient**	**MS analysis**	**Multiple Reaction Monitoring (MRM)**
Bisphenols (4 compounds)	40	0.4	(A) 10% ACN in water and (B) ACN	gradient from 10% to 90% of B in 6.5 min, then to 100% B at 7.5 min. The initial conditions were restored at 8 min, followed by a re-equilibration time of 2 min. Total run time: 10 min.	negative electrospray ionization. MS method: capillary voltage was 2.5 kV, desolvation T 650°C, cone gas 150 L/h, desolvation gas flow (N_2) 1000 L/h, and collision gas flow (Ar) 0.2 mL/min.	Detailed in Table S10
Parabens (7 compounds)	40	0.4	(A) 10% ACN in water with 0.1% of HCOOH and (B) 0.1% HCOOH in ACN	gradient from 10% to 60% of B in 6.5 min, then to 95% B at 7.5 min. The initial conditions were restored at 8 min, followed by a re-equilibration time of 2 min. Total run time: 10 min.	negative electrospray ionization. MS method: capillary voltage was 2 kV, desolvation T 650°C, cone gas 200 L/h, desolvation gas flow (N_2) 1000 L/h, and collision gas flow (Ar) 0.2 mL/min.	Detailed in Table S11
Polycyclic aromatic hydrocarbons (PAHs) (11 compounds)	35	0.4	(A) 10% ACN in water and (B) ACN	gradient from 30 % to 100% of B in 6.5 min, then to 100% B at 7.5 min. The initial conditions were restored at 8 min, followed by a re-equilibration time of 2 min. The total run time was 10 min.	positive electrospray ionization. MS method: capillary voltage was 3 kV, desolvation T 650°C, cone gas 200 L/h, desolvation gas flow (N_2) 1000 L/h, and collision gas flow (Ar) 0.19 mL/min.	Detailed in Table S12
Glyphosate and its metabolites	35	0.5	(A) 0.5% of HCOOH in	gradient from 65% to 0% of B in 3 min, then solvent composition	negative electrospray ionization. MS method: capillary voltage was 2.5 kV, desolvation T	Detailed in Table S13

(3 compounds)			water and (B) 0.5% HCOOH in ACN	remained at 100% of A for 4 min. The initial conditions were restored at 7 min, followed by a re-equilibration time of 1 min. The total run time was 8 min.	550°C, cone gas 150 L/h, desolvation gas flow (N_2) 1000 L/h, and collision gas flow (Ar) 0.18 mL/min	
Phthalates and their metabolites<br>(14 compounds)	35	0.3	(A) 10% ACN in water with 0.1% of HCOOH and (B) 0.1% HCOOH in ACN	gradient from 0% to 15% of B in 1 min, then to 40% in 6 min, to 75% of B in 13 min, and finally to 90% in 17 min. The initial conditions were restored at 18 min, followed by a re-equilibration time of 4 min. Total run time was 22 min.	(diesters): positive electrospray ionization<br>(monoesters): negative electrospray ionization<br>MS method: capillary voltage was 2 kV, desolvation T 500°C, cone gas 1500 L/h, desolvation gas flow (N_2) 1000 L/h, and collision gas flow (Ar) 0.18 mL/min.	Detailed in Table S14
Pyrethroids and chlorpyrifos<br>(3 compounds)	35	0.4	(A) 10% ACN in water and (B) ACN	gradient from 50% to 100% of B in 6.5 min, then at 100% B for 1.5 min. The initial conditions were restored at 8 min, followed by a re-equilibration time of 2 min. The total run time was 10 min.	positive electrospray ionization. MS method:<br>capillary voltage was 2.5 kV, desolvation T 450°C, cone gas 150 L/h, desolvation gas flow (N_2) 1000 L/h, and collision gas<br>flow (Ar) 0.18 mL/min.	Detailed in Table S15
Perfluoroalkyl substances (PFASs)<br>(27 compounds)	35	0.3	(A) Water +2 mM ammonium acetate and (B) MeOH +2 mM ammonium acetate	gradient from 5% to 95% of B in 14 min, then remain at 95% of B for 4 min. The initial conditions were restored at 18 min, followed by a re-equilibration time of 4 min. Total run time was 22 min.	negative electrospray ionization. MS method:<br>capillary voltage was 0.5 kV, desolvation T 350°C, cone gas 150 L/h, desolvation gas flow (N_2) 900 L/h, and collision gas flow (Ar) 0.18 mL/min.	Detailed in Table S16

Table S3. Linearity and sensitivity for each Bisphenol with the proposed analytical method

Compound	*LOD (ng/mL)*	*LOQ (ng/mL)*	*Linearity R^2*	*Curve*	*Range (ng/mL)*
Bisphenol A (BPA)	0.1	0.5	0.9978	Y = 7186.89 X - 278.06	0.1-500
Bisphenol S (BPS)	0.025	0.1	0.9954	Y = 82526.9 X - 21.1141	0.01-500
Bisphenol F (BPF)	1	10	0.9974	Y = 2107.32 X - 53.7525	0.1-500
Bisphenol F Bis(3-chloro-2-hydroxypropyl)ether (BFDGE)	1	2.5	0.9902	y = 33.1157 x + 70.3079	1-250

Table S4. Linearity and sensitivity for each Paraben with the proposed analytical method

Compound	*LOD (ng/mL)*	*LOQ (ng/mL)*	*Linearity R^2*	*Curve*	*Range (ng/mL)*
Methylparaben (MePB)	0.025	1	0.9911	Y = 432.059 X + 1.08781	0.025-250
Ethylparaben (EtPB)	0.025	0.5	0.9992	Y = 658.98 X - 2.5221	0.025-250
n-Propylparaben (PrPB)	0.1	0.5	0.9990	Y = 1315.75 X + 48.1526	0.025-250
iso-Propylparaben (iPrPB)	0.1	0.5	0.9991	Y = 758.48 X - 5.92241	0.025-250
n-Butylparaben (BuPB)	0.5	1	0.999	Y = 611.641 X - 26.5515	0.025-250
iso-Butylparaben (iBuPB)	0.1	0.5	0.999	Y = 2037.6 X + 0.546667	0.025-250
Benzylparaben (BzPB)	0.05	0.5	0.9993	Y = 1808.86 X - 29.484	0.025-250

Table S5. Linearity and sensitivity for each PAH with the proposed analytical method

Compound	*LOD (ng/mL)*	*LOQ (ng/mL)*	*Linearity R^2*	*Curve*	*Range (ng/mL)*
Anthracene (ANTHR)	1	2.5	0.9948	Y = 93.3912 X - 65.8359	5-250
Pyrene (PRY)	2.5	5	0.9910	Y = 264.037 X + 3040.16	10-250
Phenantrene (PHEN)	0.25	0.5	0.9934	Y = 42.2396 X + 174.216	5-250
Chrysene (CHRY)	0.25	0.5	0.9928	Y = 983.335 X - 297.029	0.5-250
Benz[a]antracene (BAA)	0.025	0.5	0.9916	Y = 716.457 X - 938.514	1-250
Benzo[b]fluoranthene (BBF)*	0.0025	0.025	0.9964	Y = 5385.84 X + 177.623	0.25-250
Benzo[k]fluoranthene (BKF) *	0.0025	0.025	0.9957	Y = 5370.02 X + 85.9766	0.25-250
Benzo[a]pyrene (BAP) *	0.0025	0.025	0.9953	Y = 5365.37 X + 81.0301	0.25-250
Benzo[ghi]perilene (BGHIP)	0.0025	0.05	0.9962	Y = 9310.1 X - 465.379	0.01-250
Dibenz[a,h]anthracene (DAA)	0.025	0.05	0.9952	Y = 1389.77 X + 1710.3	0.025-250
Indeno[1,2,3-cd]pyrene (IND)	0.005	0.25	0.9964	Y = 28972.1 X + 3909.47	0.2-250

*We reported the quantification of BBF, BKF, BAP as sum of the 3 analytes

Table S6. Linearity and sensitivity for each pesticide with the proposed analytical method

Compound	*LOD (ng/mL)*	*LOQ (ng/mL)*	*Linearity R^2*	*Curve*	*Range (ng/mL)*
Glyphosate (GLY)	0.1	1	0.9995	Y = 54.6574 X - 5.49079	0.1-250
Glufosinate (GLUF)	0.1	1	0.9992	Y = 193.032 X - 5.28844	0.1-250
AMPA	0.25	10	0.9994	Y = 36.4715 X - 4.86882	0.1-250

Table S7. Linearity and sensitivity for each Phthalate with the proposed analytical method

Compound	*LOD (ng/mL)*	*LOQ (ng/mL)*	*Linearity R^2*	*Curve*	*Range (ng/mL)*
Dimethyl phthalate (DMP)	0.1	0.5	0.9973	Y = 3387.07 X + 1735.6	0.01 - 500
Momomethyl phthalate (MMP)	0.05	0.1	0.9920	Y = 262457 X + 119663	0.01 - 500
Diethyl phthalate (DEP)	0.1	0.25	0.9971	Y = 12232.7 X + 4191.79	0.01 - 500
Monoethyl phthalate (MEP)	0.025	0.05	0. 9920	Y = 728012 X + 148792	0.01 - 500
Dibutyl phthalate (DBP)	0.1	0.5	0.9985	Y = 5326.3X + 355.011	0.01 - 500
Mono-*n*-butyl phthalate (MBP)	0.05	0.5	0.9987	Y = 1299.36 X + 543762	0.01 - 500
Butylbenzyl phthalate (BBP)	10	25	0.9520	Y = 72770.6 X + 2796.11	0.01 - 500
Monobenzyl phthalate (MBzP)	0.025	0.1	0.9864	Y = 2119.33 X + 269969	0.01 - 500
Di(2-ethylhexyl) phthalate (DEHP)	0.5	1	0.9978	Y = 8052.16 X + 3713.1	0.01 - 500
Mono(2-ethylhexyl) phthalate (MEHP)	0.05	0.1	0.9860	Y = 6200.48 X + 380159	0.01 - 500
Di-*n*-octyl phthalate (DNOP)	0.25	1	0.9970	Y = 10272.4 X + 1331.53	0.01 - 500
Mono-*n*-octyl phthalate (MNOP)	0.025	1	0.9891	Y = 177339 X + 315114	0.01 - 500
Mono(2-ethyl-5-hydroxyhexyl) phthalate (MEHHP)	0.05	0.1	0.9999	Y = 371.572 X + 7792.66	0.01 - 500
Mono(2-ethyl-5-oxohexyl) phthalate (MEOHP)	0.025	1	0.9826	Y = 5470.51 X + 364.284	0.01 - 500

Table S8. Linearity and sensitivity for each Pyrethroid with the proposed analytical method

Compound	*LOD (ng/mL)*	*LOQ (ng/mL)*	*Linearity R^2*	*Curve*	*Range (ng/mL)*
Cypermethrin (CP)	0.25	1	0.9990	Y = 583.827 X + 185.732	0.25-100
Cyfluthrin (CYFL)	2.5	10	0.9994	Y = 17.7698 X + 52.4514	1-250
Chlorpyrifos (CPS)	1	2.5	0.9992	Y = 126.178 X + 72.5196	0.1-250

Table S9. Linearity and sensitivity for each PFAS with the proposed analytical method

Compound	*LOD (pg/mL)*	*LOQ (pg/mL)*	*Linearity R^2*	*Curve*	*Range (pg/mL)*
PFBA	50	250	0.9991	Y = 822.006 X – 3.76771	0.05-2000
PFPeA	25	125	0.9990	Y = 1116.19 X – 5.76051	0.05-2000
PFHxA	31	125	0.9989	Y = 4290.99 X - 3.56762	0.05-2000
PFPHpA	25	63	0.9990	Y = 7443.15 X + 22.457	0.05-2000
PFOA	5	31	0.9995	Y = 5775.84 X + 179.589	0.05-2000
PFNA	25	125	0.9990	Y = 8881.77 X + 64.5032	0.05-2000
PFDA	25	31	0.9991	Y = 6704.04 X + 16.0629	0.05-2000
PFUnDA	50	125	0.9992	Y = 4252.04 X – 14.7561	0.05-2000
PFDoDA	25	31	0.9994	Y = 5610.79 X - 4.39419	0.05-2000
PFTrDA	25	63	0.9992	Y = 594.833 X – 2.92044	0.05-2000
PFTreDA	25	63	0.9991	Y = 374.151 X + 4.10484	0.05-2000
PFBS	5	63	0.9994	Y = 4199.02 X – 52.5595	0.05-2000
PFPeS	2.5	125	0.9990	Y = 4285.46 X – 64.5152	0.05-2000
PFHxS	25	63	0.9990	Y =3036.42 X – 22.5271	0.05-2000
PFHpS	2.5	31	0.9993	Y = 3126.82 X – 30.0789	0.05-2000
PFOS	25	125	0.9990	Y = 2472.13 X + 14.8939	0.05-2000
PFNS	0.5	15	0.9991	Y = 2551.39 X – 12.5212	0.05-2000
PFDS	5	63	0.9991	Y = 1925.38 X + 19.0492	0.05-2000
GenX (HFPO-DA)	250	1000	0.9915	Y = 122.232 X – 21.2713	0.05-2000
ADONA	2.5	15	0.9993	Y = 19311.6 X – 152.379	0.05-2000
9Cl-PF3ONS	2.5	31	0.9994	Y = 15586.7 X – 44.2447	0.05-2000
11Cl-PF3OUdS	2.5	15	0.9995	Y = 10095.6 X + 96.3466	0.05-2000
4 :2 FTS	5	63	0.9991	Y = 2933.86 X – 39.5514	0.05-2000
6:2 FTS	25	63	0.9990	Y = 2910.83 X – 37.9872	0.05-2000
8 :2 FTS	2.5	250	0.9980	Y = 2588.53 X – 55.2677	0.05-2000
FOSA	2.5	31	0.9991	Y = 7438.6 X – 7.34504	0.05-2000
FBSA	2.5	31	0.9994	Y = 8556.6 X – 60.0129	0.05-2000

Table S10. Analytical Rt and MRM transition parameters of bisphenols

Chemical compound	*Abbreviations*	*Rt (min)*	*ESI mode*	*Precursor m/z*	*Transition m/z*	*Cone voltage (V)*	*Collision energy (eV)*
Bisphenol A	BPA	2.95	(-)	227	(QN) 212	30	26
4,4'-(1-Methylethylidene)bisphenol					(QL) 133		18
Bisphenol S	BPS	1.90	(-)	249	(QN) 108	30	40
4,4'-Sulfonylbisphenol					(QL) 92.0		30
Bisphenol F	BPF	2.46	(-)	199	(QN) 105	30	15
4,4'-Methylenebisphenol					(QL) 93.0		20
Bisphenol F Bis(3-chloro-2-hydroxypropyl)ether	BPFDGE	3.98	(-)	402	(QN) 199	30	20
1-chloro-3-[4-[[4-(3-chloro-2-hydroxypropoxy)phenyl]methyl]phenoxy]propan-2-ol					(QL) 181.3		10

Table S11. Analytical Rt and MRM transition parameters of Parabens

Chemical compound	*Abbreviations*	*Rt (min)*	*ESI mode*	*Precursor m/z*	*Transition m/z*	*Cone voltage (V)*	*Collision energy (eV)*
Methylparaben	MePB	2.35	(-)	151	(QN) 92	30	15
Methyl 4-hydroxybenzoate					(QL) 136		15
Ethylparaben	EtPB	3.03	(-)	165	(QN) 92	30	20
Ethyl 4-hydroxybenzoate					(QL) 93		20
n-Propylparaben	PrPB	3.79	(-)	179	(QN) 92	30	25
Propyl 4-hydroxybenzoate					(QL) 136		18
iso-Propylparaben	iPrPB	3.64	(-)	179	(QN) 137	27	21
Propan-2-yl 4-hydroxybenzoate					(QL) 93.0	30	13
n-Butylparaben	BuPB	4.48	(-)	193	(QN) 93.0	31	21
Butyl 4-hydroxybenzoate					(QL) 137		17
iso-Butylparaben	iBuPB	4.39	(-)	193	(QN) 92.0	30	23
2-Methylpropyl 4-hydroxybenzoate					(QL) 136		20
Benzylparaben	BzPB	4.93	(-)	227	(QN) 136	27	15
Propyl 4-hydroxybenzoate					(QL) 92.0		17

Table S12. Analytical Rt and MRM transition parameters of PAHs

Chemical compound	*Abbreviations*	*Rt (min)*	*ESI mode*	*Precursor m/z*	*Transition m/z*	*Cone voltage (V)*	*Collision energy (eV)*
Anthracene	ANTHR	2.35	(+)	178	(QN) 152	30	30
Anthracene					(QL) 176		45
Pyrene	PYR	3.90	(+)	202	(QN) 202	30	45
Pyrene					(QL) 200		20
Phenanthrene	PHEN	2.35	(+)	178	(QN) 176	30	50
Phenanthrene					(QL) 151		40
Chrysene	CHRY	4.26	(+)	228	(QN) 226	30	35
Chrysene					(QL) 202		38
Benz[a]antracene	BAA	4.34	(+)	228	(QN) 226	30	35
Tetraphene					(QL) 202		45
Benzo[b]fluoranthene	BKF	4.70	(+)	252	(QN) 250	30	35
Benzo[e]acephenanthrylene					(QL) 226		50
Benzo[a]pyrene	BAP	2.77	(+)	252	(QN) 250	30	35
Benzo[pqr]tetraphene					(QL) 226		50
Benzo[ghi]perylene	BGHIP	5.11	(+)	276	(QN) 274	30	70
Benzo[ghi]perylene					(QL) 248		55
Dibenz[a,h]anthracene	DAA	4.95	(+)	278	(QN) 226	30	52
Benzo[k]tetraphene					(QL) 202		20
Indeno[1,2,3-cd]pyrene	IND	5.11	(+)	276	(QN) 276	30	52
Indeno[1,2,3-cd]pyrene					(QL) 274		20

Table S13. Analytical Rt and MRM transition parameters of glyphosate and its metabolites

Chemical compound	*Abbreviations*	*Rt (min)*	*ESI mode*	*Precursor m/z*	*Transition m/z*	*Cone voltage (V)*	*Collision energy (eV)*
Glyphosate	GLY	5.82	(-)	168	?	20	24
2-(Phosphonomethylamino)acetic acid					(QL) 79		29
Glufosinate	GLUF	2.81	(-)	180	?	30	24
2-Amino-4-[hydroxy(methyl)phosphoryl]butanoic acid					(QL) 95		23
AMPA	AMPA	1.05	(-)	110		30	16
(Aminomethyl)phosphonic acid					(QL) 81		10

Table S14. Analytical Rt and MRM transition parameters for phathalates

Chemical compound	*Abbreviations*	*Rt (min)*	*ESI mode*	*Precursor m/z*	*Transition m/z*	*Cone voltag e (V)*	*Collision energy (eV)*
Dimethyl phthalate<br>*Dimethyl benzene-1,2-dicarboxylate*	DMP	3.36	(+)	195	(QN) 163<br>(QL) 133	15	30<br>15
Monomethyl phthalate<br>*2-(Methoxycarbonyl)benzoic acid*	MMP	2.57	(-)	178.9	(QN) 77.0<br>(QL) 107	30	17<br>10
Diethyl phthalate<br>*Diethyl benzene-1,2-dicarboxylate*	DEP	4.36	(+)	223	(QN) 149<br>(QL) 177	20	19<br>10
Monoethyl phthalate<br>*2-(Ethoxycarbonyl)benzoic acid*	MEP	3.11	(-)	193	(QN) 77.0<br>(QL) 121	30	15<br>11
Dibutyl phthalate<br>*Dibutyl benzene-1,2-dicarboxylate*	DBP		(+)	279	(QN) 149<br>(QL) 205	13	14<br>7
Mono-*n*-butyl phthalate<br>*2-(Butoxycarbonyl)benzoic acid*	MBP	4.25	(-)	221	(QN) 77.0<br>(QL) 71.0	30	14<br>16
Butylbenzyl phthalate<br>*2-O-benzyl 1-O-butyl benzene-1,2-dicarboxylate*	BBP	7.17	(+)	313	(QN) 91<br>(QL) 149	23	22<br>14
Monobenzyl phthalate<br>*2-[(Benzyloxy)carbonyl]benzoic acid*	MBzP	4.80	(-)	225	(QN) 183<br>(QL) 107	30	14<br>11
Di(2-ethylexyl) phthalate<br>*Di(2-ethylhexyl)benzene-1,2-dicarboxylate*	DEHP	8.12	(+)	391.7	(QN) 113.4<br>(QL) 149	33	19<br>11
Mono(2-ethylexyl) phthalate<br>*2-(2-Ethylhexoxycarbonyl)benzoic acid*	MEHP	6.08	(-)	277	(QN) 134<br>(QL) 77	30	19<br>16
Di-*n*-octyl phthalate<br>*Dioctyl benzene-1,2-dicarboxylate*	DNOP	8.12	(+)	391	(QN) 149<br>(QL) 261.2	30	16<br>10
Monooctyl phthalate<br>*2-(Octoxycarbonyl)benzoic acid*	MnOP	6.1	(-)	277	(QN) 77.0<br>(QL)125	30	29<br>23
Mono(2-ethyl-5-hydroxyhexyl) phthalate<br>*2-(2-Ethyl-5-hydroxyhexoxycarbonyl)benzoic acid*	MEHHP	4.2	(-)	294	(QN) 121<br>(QL) 145	30	20<br>15
Mono(2-ethyl-5-oxohexyl) phthalate<br>*2-(2-Ethyl-5-oxohexoxycarbonyl)benzoic acid*	MEOHP	4.46	(-)	291	(QN) 143<br>(QL) 121	30	19<br>14

Table S15. Analytical Rt and MRM transition parameters for pyrethroids and chlorpyrifos

Chemical compound	*Abbreviations*	*Rt (min)*	*ESI mode*	*Precursor m/z*	*Transition m/z*	*Cone voltage (V)*	*Collision energy (eV)*
Cypermethrin	CP	3.32	(+)	181	(QN) 152	30	20
[cyano-(3-phenoxyphenyl)methyl] 3-(2,2-dichloroethenyl)-2,2-dimethylcyclopropane-1-carboxylate				163	(QL) 127		10
Cyfluthrin	CYFL	3.2	(+)	163	(QN) 91	30	25
[cyano-(4-fluoro-3-phenoxyphenyl)methyl] 3-(2,2-dichloroethenyl)-2,2-dimethylcyclopropane-1-carboxylate				206	(QL) 151		19
Chlorpyrifos	CPS	2.62	(+)	351.9	(QN) 124.9	30	17
Diethoxy-sulfanylidene-(3,5,6-trichloropyridin-2-yl)oxy-λ5-phosphane					(QL) 199		20

Table S16. Analytical Rt and MRM transition parameters for PFAS

Chemical compound	*Abbreviations*	*Rt (min)*	*ESI mode*	*Precursor m/z*	*Transition m/z*	*Cone voltage (V)*	*Collision energy (eV)*
Perfluoro-n-butanoic acid	PFBA	3.92	(-)	213.0	(QN) 169	8	5
Perfluoro-n-pentanoic acid	PFPeA	6.36	(-)	262.9	(QN) 218.9	5	5
Perfluoro-n-hexanoic acid	PFHxA	8.30	(-)	312.9	(QN) 268.9<br>(QL) 118.9	16<br>16	6<br>21
Perfluoro-n-heptanoic acid	PFPHpA	9.72	(-)	362.9	(QN) 318.9<br>(QL) 168.9	14<br>14	8<br>14
Perfluoro-n-octanoic acid	PFOA	10.84	(-)	412.9	(QN) 368.9<br>(QL) 168.9	22<br>22	7<br>15
Perfluoro-n-nonanoic acid	PFNA	11.76	(-)	462.9	(QN) 418.9<br>(QL) 218.9	18<br>18	9<br>15
Perfluoro-n-decanoic acid	PFDA	12.55	(-)	512.9	(QN) 468.9<br>(QL) 218.9	6<br>6	9<br>15
Perfluoro-n-undecanoic acid	PFUnDA	13.23	(-)	562.9	(QN) 518.9<br>(QL) 268.9	8<br>8	8<br>14
Perfluoro-n-dodecanoic acid	PFDoDA	13.82	(-)	612.9	(QN) 568.9<br>(QL) 168.9	12<br>12	12<br>21
Perfluoro-n-tridecanoic acid	PFTrDA	14.33	(-)	662.9	(QN) 168.9<br>(QL) 218.9	14<br>14	22<br>20
Perfluoro-n-tetradecanoic acid	PFTreDA	14.76	(-)	712.9	(QN) 218.9<br>(QL) 168.9	14<br>14	22<br>20
Perfluoro-1 butanesulphonamide	PFBS	6.88	(-)	298.9	(QN) 80.1<br>(QL) 99.1	7<br>7	27<br>27
Perfluoropentanesulphonic acid	PFPeS	8.55	(-)	348.9	(QN) 79.9<br>(QL) 98.9	32<br>32	31<br>25
Perfluorohexanesulphonic acid	PFHxS	9.83	(-)	398.9	(QN) 80.1<br>(QL) 99.1	38<br>38	35<br>29
Perfluoroheptanesulphonic acid	PFHpS	10.88	(-)	448.9	(QN) 79.9<br>(QL) 98.9	16<br>16	34<br>34
Perfluorooctanesulphonic acid	PFOS	11.76	(-)	498.9	(QN) 79.9<br>(QL) 98.9	30<br>30	42<br>40
Perfluorononanesulfonic acid	PFNS	12.52	(-)	548.9	(QN) 80.1<br>(QL) 99.1	24<br>24	40<br>36
Perfluorododecanenesulfonic acid	PFDS	13.19	(-)	598.9	(QN) 80.1<br>(QL) 99.1	46<br>46	46<br>46
2,3,3,3-tetrafluoro-2-(heptafluoropropoxy)propanoic acid	GenX (HFPO-DA)	8.78	(-)	285.0	(QN) 169<br>(QL) 119	5	7<br>5
4,8-Dioxa-3H-perfluorononanoic acid	ADONA	9.86	(-)	376.9	(QN) 251<br>(QL) 84.9	12<br>10	10<br>12
Perfluoro(2-((6-chlorohexyl)oxy)ethanesulfonic acid)	9Cl-PF3ONS	12.24	(-)	531.0	(QN) 351<br>(QL) 82.9	14<br>14	22<br>20
11-chloroeicosafluoro-3-oxaundecane-1-sulfonic acid	11Cl-PF3OUdS	13.55	(-)	631.0	(QN) 450.9<br>(QL)82.9	16<br>16	26<br>16
3,3,4,4,5,5,6,6,6-nonafluorohexane-1-sulfonic acid	4 :2 FTS	8.12	(-)	326.9	(QN) 306.9<br>(QL) 80.9	42<br>18	18<br>42
3,3,4,4,5,5,6,6,7,7,8,8,8-tridecafluorooctane-1-sulfonic acid	6:2 FTS	8.12	(-)	427.0	(QN) 406.9<br>(QL) 80.1	12	22<br>12
3,3,4,4,5,5,6,6,7,7,8,8,9,9,10,10,10-heptadecafluorodecane-1-sulfonic acid	8 :2 FTS	13.53	(-)	526.9	(QN) 506.9<br>(QL) 80.9	28<br>26	28<br>26
Perfluorobutane Sulfonamide	FBSA	8.64	(-)	297.9	(QN) 78<br>(QL)118.9	25<br>25	25<br>15
Perfluorooctanesulfonamide	FOSA	13.33	(-)	498.0	(QN) 77.9	40	29

Table S17. Linearity and sensitivity for each metal and metalloid measured by ICP-AES

Element	*LOD (ng/mL)*	*LOQ (ng/mL)*	*Linearity R^2*	*Curve*	*Range (ng/mL)*
Al	0.2	0,5	0,9999	y = 40 x + 184	1-100
As	0,4	1	0.9999	y = 17.7698 x + 52.4514	1-50
Ba	0.2	0,5	0.9998	y = 1248 x + 477	1-100
Bi	0.2	0,5	0.9999	y = 2.0 x + 7.1	1-50
Cd	0,02	0,05	0,9999	y = 32.6 x + 21.3	1-50
Co	0.2	0,5	0,9989	y = 9.04 x + 17.5	1-50
Cr	0.2	0,5	0,9997	y = 23.3 x + 17.1	1-50
Cu	0.2	0,5	0,9999	y = 7.85 x + 13.0	1-100
Mn	0.2	0,5	0,9999	y = 256.9 x + 100.5	1-100
Mo	0.2	0,5	0,9997	y = 3.24 x + 8.1	1-100
Ni	0.2	0,5	0,9935	y = 5.34 x + 13.5	1-50
Pb	0.2	0,5	0,9964	y = 0.51 x + 18.8	1-50
Ti	0.2	0,5	0,9999	y = 220.3 x + 236	1-50
Tl	0.2	0,5	0,9965	y = 0.72 x + 5.79	1-50
V	0.2	0,5	0,9998	y = 25.34 x + 31.5	1-50
Zn	0,4	1	0,9999	y = 31.1 x + 348	1-100
